# Improving the foundation for particulate matter risk assessment by individual nanoparticle statistics from electron microscopy analysis

**DOI:** 10.1038/s41598-019-44495-7

**Published:** 2019-05-30

**Authors:** Anders Brostrøm, Kirsten Inga Kling, Ismo Kalevi Koponen, Karin Sørig Hougaard, Konrad Kandler, Kristian Mølhave

**Affiliations:** 1 National Centre for Nano Fabrication and Characterization (DTU Nanolab), Technical University of Denmark, 2800 Kgs. Lyngby, Denmark; 2 Metrology and Air Environment, Force Technology, 2605 Brøndbyvester, Denmark; 3 National Research Centre for the Working Environment, 2100 Copenhagen, Denmark; 4 Institut für Angewandte Geowissenschaften, Technical University of Darmstadt, 64287 Darmstadt, Germany

**Keywords:** Characterization and analytical techniques, Regulation and risk management, Nanoparticles

## Abstract

Air pollution is one of the major contributors to the global burden of disease, with particulate matter (PM) as one of its central concerns. Thus, there is a great need for exposure and risk assessments associated with PM pollution. However, current standard measurement techniques bring no knowledge of particle composition or shape, which have been identified among the crucial parameters for toxicology of inhaled particles. We present a method for collecting aerosols via impaction directly onto Transmission Electron Microscopy (TEM) grids, and based on the measured impactor collection efficiency and observed impact patterns we establish a reproducible imaging routine for automated Scanning Electron Microscopy (SEM) analysis. The method is validated by comparison to scanning mobility particle sizer (SMPS) measurements, where a good agreement is found between the particle size distributions (PSD), ensuring a representative description of the sampled aerosol. We furthermore determine sampling conditions for achieving optimal particle coverage on the TEM grids, allowing for a statistical analysis. In summary, the presented method can provide not only a representative PSD, but also detailed statistics on individual particle geometries. If coupled with Energy-dispersive X-ray spectroscopy (EDS) analysis elemental compositions can be assessed as well. This makes it possible to categorize particles both according to size and shape e.g. round and fibres, or agglomerates, as well as classify them based on their elemental composition e.g. salt, soot, or metals. Combined this method brings crucial knowledge for improving the foundation for PM risk assessments on workplaces and in ambient conditions with complex aerosol pollution.

## Introduction

Air pollution is a growing issue, now recognized as one of the major contributors to the global burden of disease, with particulate matter (PM) as one of the central concerns^1^. Particles are released from a wide range of both natural and anthropogenic sources, where especially the transport sector is a major contributor in the urban environment^2^. The main indoor sources include cooking, smoking, and cleaning with sprays or vacuum cleaners^3^. In industrial settings examples of particle sources with high exposures include powder handling, sanding, spray painting, welding, and combustion processes^4,5^. Additionally the use of nano materials in every day products is increasing rapidly and nanosized particles are now common constituents in for example pharmaceuticals, cosmetics, electronics, and food packaging^6–8^, leading to new exposure scenarios during production, use, and disposal^9^. Exposure to particles for workers, users, and the general public is therefore evident; however the risks associated with exposure from the different sources are not trivial to evaluate, due to the complex nature of aerosols as well as their adverse health effects.

Risk is related to exposure, and the main exposure route for particulate matter is via the respiratory system; with the aerodynamic size governing where in the respiratory system the particles are most likely to deposit^10^. Particles larger than 1 *μ*m deposit mainly in the upper airways, whereas ultrafine, sub 1 *μ*m particles, and nanoparticles are more likely to reach the deep parts of the lungs, depositing in the tracheobronchial and alveolar regions^11^.

Current exposure limits in legislation are mass based, which in the past has proven a useful and simple metric for associating particle levels with a wide range of health outcomes; however, there are also many cases where mass has been found insufficient for assessing risk. These cases include situations where a low PM mass results in high risk, for example if particles consist of biologically potent materials e.g. transition metals, or if the particle population is dominated by a large number of nanoparticles with a limited contribution to the overall mass^12^. Cases also occur where the PM mass is dominated by low-toxicity components such as solvable salts or crustal dust posing little risk to human health^13^. It is therefore well accepted that mass is not an ideal metric for risk assessments, and at best is only a rough indicator of risk. In fact, studies have shown that the particle number or total particle surface area, which is not necessarily related to mass, can be better descriptors for e.g. inflammatory and oxidative stress responses^14,15^. It has furthermore been recognized that particle shape, especially in the case of fibres, is an important parameter for determining toxicological effects upon inhalation^16,17^. Assessing risk of PM hence requires detailed information of the particle size distribution (PSD).

There are currently many instruments available to characterize PSDs. Some of the most common include Electric Low Pressure Impactors (ELPI), Diffusion Chargers (DC), Scanning Mobility Particle Sizers (SMPS), Nanoparticle Surface Area Monitors (NSAM), Condensation Particle Counters (CPC), and Optical particles Sizers (OPS)^18,19^. These instruments measure either or both the number and diameter of particles, with a high resolution in both size and time, giving real-time data within a few seconds or minutes. All the instruments operate in specific size regimes within the nanometre to micrometer range. Some instruments give additional information e.g. estimates of total particle surface area, lung deposited surface area (LDSA), or mass concentration, assuming spherical particles with a fixed density. The mass of an aerosol may also be determined by weighing a filter or a stage in a controlled environment both before and after pulling air through the filter. Here larger particles can be removed prior to sampling using an impactor or a cyclone. By combining the different measurement techniques it is therefore possible to characterize a PSD in terms of size, number, and mass, as well as to obtain estimates of surface area and LDSA. However, none of the mentioned instruments give information on particle morphology or composition. It is therefore not possible to distinguish between different types of particles as fibres/agglomerates or between particles from different sources. New and additional measurement techniques are therefore needed to give a more detailed description of aerosol populations in terms of size, number, shape, composition, and surface area. This will enable establishment of standard procedures for quantifying and regulating particulate exposure and the associated health risks. Such individual particle statistics will help discriminate between particle origins and hence facilitate a better understating of the sources and their health risks, bringing crucial knowledge for the development of preventive strategies.

Electron microscopy (EM) is an analysis technique, which allows visualization of particles down to the nanometre scale, thereby providing physical information on the single particle level e.g. geometric size, agglomeration state, and shape^20,21^. EM can furthermore measure the elemental composition of individual particles when combined with energy dispersive x-ray spectroscopy (EDS or EDX), enabling a more detailed description of aerosol populations^22,23^ than the real time instruments.

The most common EM techniques are Transmission Electron Microscopy (TEM) and Scanning Electron Microscopy (SEM), where TEM allows for the highest resolution, down to Angstrom scales. Transmission electron microscopy analysis is however a time consuming process for obtaining statistically robust data on shape, composition, and size distributions of aerosol populations. Furthermore the high TEM resolution is often not required for aerosol particles larger than a few tens of nanometres. Scanning electron microscopes provides a lower resolutions, but recent advances have made it possible to automatically scan large areas of a sample, process the acquired images, locate particles, and perform subsequent EDS analysis without user intervention except for the initial setup^24,25^. As a result, automated SEM/EDS analysis makes it possible to obtain sufficient data on the single particle level to allow for a meaningful statistical analysis of an aerosol population, including both physical and elemental composition data^26^. Electron microscopy is however, an offline measurement technique and therefore only provides detailed information some time after sampling. It should therefore be used in combination with real time instruments, enabling online number and size data along with a more detailed characterization from automated SEM/EDS. Real time instruments are furthermore useful to estimate sampling times for achieving reasonable particle densities on collected samples, and are necessary to identify crucial processes, where sampling is relevant, e.g. peak emissions of industrial processes, or at relevant times of day in the ambient environment.

In order to use SEM it is necessary to collect the airborne particles onto a surface. There are currently many collection methods e.g. thermophoretic precipitation^27^, filtering, electrostatic collection, and impaction^28,29^. Each method collects the particles onto a surface, where common choices include aluminum foil, silver foil^30^, polyurethane foams, Teflon filters, quartz fibre filters^31^, and TEM grids of different design and substrate type. The samples can then either be inserted directly into the electron microscope or may need additional steps before analysis. For some filters the preparatory steps involve dissolving the particles in a liquid and transferring them to a different surface, which is better suited for imaging^32^. The steps may also include drying or freezing to stabilize or maintain specific particle characteristics in the vacuum of the microscope^33^, or applying a conductive coating to avoid charging under the electron beam^34^. However, it is generally undesirable to have preparatory steps, since they may alter the state or appearance of the particles before analysis^35^. An additional consideration is the sampling time, since it governs the particle load on the sample. If the sampling time is too short, it will not be possible to conduct a statistical analysis on the few particles, while sampling too long results in particle co-deposition making it difficult to distinguish individual particles and assess if they were airborne as agglomerates or single primary particles. In literature, the broad range of collection techniques, substrates, and filters make it difficult to compare samples and results, as there are currently no available standard operating procedures ensuring a representative and reproducible description of a sampled aerosol population.

The aim of this work is to develop a standard operating procedure for collecting airborne particles directly onto Formvar/carbon coated TEM grids using a three stage cascade impactor^36^. The impaction technique allows for short sampling times, typically 5–30 seconds, while the TEM grids provide a conductive, thin, and uniform substrate. This eases the image processing, minimizes charge effects under the electron beam, and allows for direct analysis by both SEM and TEM without any preparatory steps. In this work we focus on the impactor stage that samples the smallest diameter particles from approximately 50 to 600 nm, which is in the size regime that pose the greatest challenge for current legislation due to their low contribution to the overall particle mass. The impactor stage cut-off diameter (D_50_) and an expression for the collection efficiency (C_eff_) curve are determined using spherical polystyrene particles with unit density. The collected aerosol samples are investigated by SEM in order to visualize the impaction patterns, which are compared to published computational and experimental studies of particle deposition patterns in tapered circular jets of impactors^37,38^. Using the knowledge of the impaction pattern an imaging routine is established. The PSD resulting from the imaging routine is compared to SMPS measurements, ensuring a reproducible and representative sample description. Tests are furthermore conducted to estimate an optimal sampling time as a function of particle number concentration by minimizing co-deposition. Finally the method is overall compared to data obtainable with other standard methods.

## Materials and Methods

### Impactor

**Figure 1:**
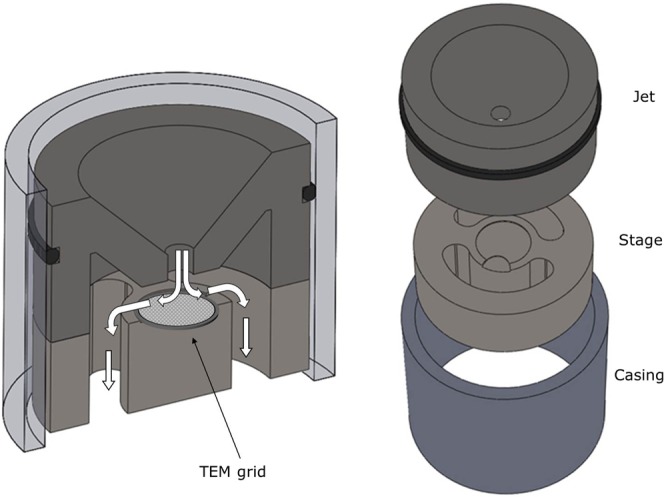
Drawings of a stage and jet of the MINI impactor. Views are presented as assembled and exploded for easy visualization.

Particles were collected with the Micro INertial Impactor (MINI)^36^, which consists of three stages that can each be equipped with TEM grids. Drawings of one of the stages equipped with a TEM grid is shown in Fig. 1. The impactor has tapered round nozzles with diameters 1, 0.6, and 0.29 mm, and jet-to-plate distance ratios (S/W) of 1.3, which fulfills the design criteria described by^39^ and^40^. A cross sectional view of the impactor design and the design parameters can be found in the SI.

A diaphragm gas pump model NMP 830 (KNF Neuberger, Germany) was used for sampling, resulting in a flow rate of 0.76 L min^−1^ through the impactor, measured with a Gilian Gilibrator-2 (Sensidyne, USA). At ambient conditions this yields theoretical D_50_ of 1.36, 0.59, and 0.055 *μ*m^41^ and Reynold numbers of 1030, 1710, and 3320 for the three stages, respectively. The Reynolds numbers are therefore within or close to the 500–3000 range ensuring sharp collection efficiency curves at the cut-off sizes^42^. In this work only the final stage of the impactor was studied, equipped with 400 mesh nickel TEM grids coated with a 25–50 nm Formvar film with 1 nm carbon deposited on top (Electron Microscopy Sciences (EMS), USA). Thinner Formvar options were tested but these often resulted in large areas with broken film and are therefore not recommended. The 400 mesh option was chosen as it offered the smallest areas of unsupported film and was thought to stabilize the substrate most efficiently, while the thin carbon layer was found sufficient to avoid charging effects under the electron beam. In this study London finder grids were used, which offer unique markings on the grids for easy orientation; however, these marking were often found to interfere with the patterns in the impact areas, and it is therefore recommended to use grids with a simple and regular design instead. Nickel grids were chosen as they are magnetic and can be held in place with magnets, which were inserted into the impactor stages from the bottom. This ensures minimal movement of the grid during sampling.

### Electron microscopy and image analysis

Electron microscopy samples were analyzed with a Nova NanoSEM 600 (FEI, The Netherlands), equipped with an OPTIMUS TKD detector (Bruker, Germany), functioning as a scanning transmission electron microscopy (STEM) detector. The STEM detector gives a high contrast between substrate and particles, and was found more efficient than the traditional secondary electron (SE) detector for visualizing the small low contrast particles. Since this eases the image segmentation, only images from the STEM detector were used for analysis. The microscope was operated in high vacuum mode with acceleration voltages of 10–20 keV and a probe current of 12 nA. Overview images were acquired at magnifications of 500–1000, while image sequences were all performed at 20 k magnification, corresponding to a resolution of 3.7 nm/pixel. The Esprit software (Bruker, Germany) was used for automated image acquisition procedures.

Images were analyzed using a Python script with the OpenCV package^43^, where a manually set global or mean adaptive threshold was used for segmentation. A more detailed description of the segmentation procedure and the determination of a particle detection limit from the images are described in the SI.

### Setup for measuring collection efficiency

**Figure 2:**
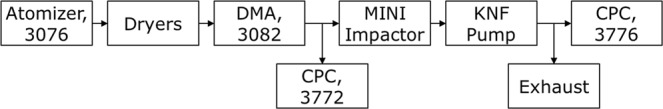
Schematic of the experimental setup for determining impactor collection efficiency.

A setup was designed to experimentally determine the C_eff_ curve and D_50_ for the lowest stage of the MINI impactor. The D_50_ value for the stage was theoretically determined to 55 nm, meaning that it would collect the majority of particles in the ultrafine and nano regimes, since the D_50_ of the middle stage was determined to 590 nm. The experimental setup is shown in Fig. 2.

The setup consisted of a constant output atomizer model 3076 (TSI, USA), operated at a back pressure of 2 bars. The atomizer generated aerosols of spherical polystyrene latex beads (PSL) from a solution made by mixing 2 drops of 1 wt% solutions (Bangs Laboratoriets inc., USA) of 42 ± 0.5 nm, 75.8 ± 1, 102.7 ± 1.3, 150 ± 1.9, and 207 ± 2.6 nm into 100 ml of nanopure water with a resistivity of 18 MΩ.cm. The solution was sonicated for 5 minutes prior to use, in order to minimize agglomeration. From the atomizer, the aerosol was passed through two silica dryers, decreasing the relative humidity from 100% to 16%, which slowly increased to 20% during experiments. A differential mobility analyzer (DMA) model 3082 (TSI, USA) was operated statically to generate a monodisperse aerosol from the dried aerosol flow, at a sample flow rate of 1.79 l/min with a sheath flow of 17.9 l/min. To measure the collection efficiency as function of size, a series of experiments were conducted where the DMA produced monodisperse aerosols from 20 to 200 nm in steps of 10 nm. The number concentration of the monodisperse aerosol was determined after the DMA with a CPC model 3772 (TSI, USA), while another part of the aerosol was sampled with the impactor, followed by a CPC model 3776 (TSI, USA) operated at low flow mode (0.3 l/min). Polyvinyl chloride (Tygon™) tubing (ID = 4.8 mm) was used for all connections to minimize electrostatic losses^44^. Tube lengths from the DMA to the CPC and MINI impactor were kept as short as possible with lengths of 25 and 27 cm respectively. Prior to experiments the two CPCs were tested in parallel, where they showed similar readings allowing direct comparison between them. Excess air after the impactor was led to an exhaust. At each DMA setting the CPCs measured for a minimum of two minutes. The fraction of particles removed was determined from the ratio between the number concentration before and after the impactor. The impactor was operated both with and without a TEM grid collection plate. The configuration without a collection plate was used to investigate the overall number of particles lost due to wall deposition in the flow path, impactor, and inside the KNF pump. It was not possible to separate the three loss contributions, and therefore only the overall loss of particles was determined. The configuration with a collection plate was used to determine the number of particles impacting on the TEM grid. It was assumed that losses to walls, the orifice, and the pump were the same for both configurations, making it possible to calculate C_eff_ as the difference between the fractional removals. A test was also performed with a TEM grid stage installed under the 0.6 mm orifice followed by the critical 0.25 mm orifice, which was installed to maintain the same flow conditions. The test was performed to investigate if the sub 590 nm size distribution was affected by the higher stages. The results were similar to the case without a collection plate installed, showing minimal influence from the higher stages.

### Setup for aerosol characterization

**Figure 3:**
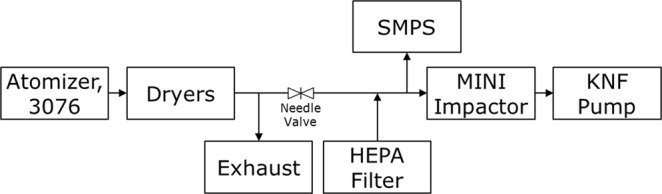
Schematic of the experimental setup used to generate a poly disperse aerosol for investigating impaction patterns and optimal sampling times.

A rearranged version of the previous setup was used for determining the optimal sampling time and for investigating the impaction pattern on the TEM grids. A schematic of the setup is shown in Fig. 3.

The aerosol was diluted with HEPA filtered room air. The degree of dilution was regulated with a needle valve, controlling the flow from the original aerosol, with excess going to an exhaust. Part of the diluted aerosol was led to the DMA and CPC model 3776, which was operated as a SMPS with a sample flow of 0.3 l/min and a sheath flow of 3.0 l/min. These settings allowed for scans ranging from 17.5 to 532.8 nm. The rest of the diluted aerosol was led to the impactor for particle collection. To visualize the impaction patterns for a broad range of particle sizes, an aerosol consisting of PSL beads was made from one drop of 42 ± 0.5, two drops of 102.7 ± 1.3, three drops of 207 ± 2.6, and six drops of 505 ± 6.4 nm 1 wt% PSL solutions (Bangs Laboratoriets inc., USA) into 100 ml of nanopure water. The solution was sonicated for 5 minutes before use.

## Results and Discussion

### Collection efficiency and cut-off diameter

**Figure 4:**
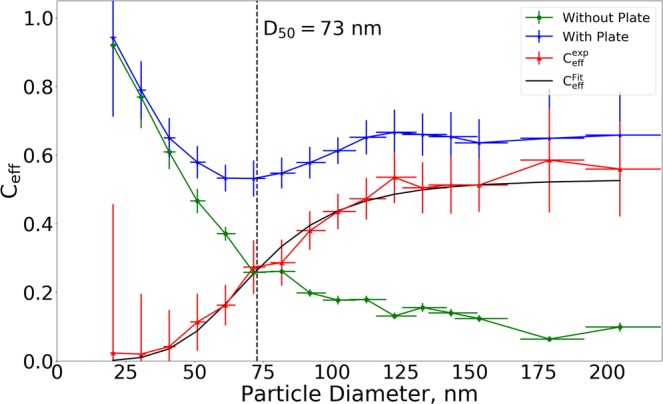
Experimentally determined collection efficiency curves for the 0.29 mm orifice both with (blue) and without (green) impaction plate installed. The difference between the two impactor configurations is plotted in red along with a fitted collection efficiency curve (black). The marked D_50_ value of 73 nm was determined from the fitted curve.

The particle number concentrations measured before and after the impactor for all selected particle sizes, both with and without collection plate, are shown in the SI. The fraction of particles removed by the impactor (C_eff_) was calculated as the ratio between 2 minute average concentrations measured before and after the impactor for each DMA selected size, as depicted in Fig. 4. Uncertainties in removal efficiency are determined from the standard deviations of the CPC measurements during the 2 minute intervals, while uncertainties in particle size are determined as the half width of the DMA transfer function^45^.

From Fig. 4 it is seen that a fraction of all sizes of particles are removed while going through the impactor both with and without a collection plate installed. For the case without a collection plate the removal is decreasing with increasing particle size. The highest removal is seen at 20 nm where 92 ± 20% of the particles disappear when going through the impactor. A lower limit of removal is reached for particles larger than approximately 120 nm, from which 10–20% of the particles disappear when going through the impactor. Since these losses occur without a collection plate installed they must be attributed to removal processes other than impaction, which are most likely wall losses, evaporation or fractioning in the jet, or losses within the KNF pump. It was not possible to separate these individual processes, while maintaining the 0.76 l/min flow through the impactor and their relative contributions are therefore not known. For the case with a collection plate installed, the highest removal is seen at 20 nm as for the case without a collection plate, indicating that similar processes are governing the removal for particles of this size. However, a significantly higher removal is observed for particles above 50 nm, which must be due to particles impacting onto the collection plate. From 70 nm and up the collection efficiency starts to increase before levelling to approximately 65% for particles larger than 120 nm. Ideally the removal should rise to 100% with all particles impacting on the collection plate, but since these are hard and relatively dry spheres (RH% = 16–20%), a significant particle bounce effect was expected^46,47^. Assuming that the losses to walls, the orifice, and the pump are equivalent for both impactor configurations, it is possible to obtain the ${{\rm{C}}}_{{\rm{eff}}}^{\exp }$ curve describing the fraction of particles impacted onto the TEM grid by subtracting the two data sets, as seen in Fig. 4. The resulting data points give a s-shaped curve, which can be fitted with the Sigmoid function shown in Equation (1) ^48^:

1 $${{\rm{C}}}_{{\rm{eff}}}^{{\rm{Fit}}}=\frac{{{\rm{c}}}_{{\rm{\max }}}}{1+{\frac{{D}_{50}}{d}}^{2s}}$$

where d is a given particle diameter, s is a fitted steepness parameter, and c_mac_ is the asymptotic collection efficiency reached at particle sizes much larger than D_50_, which theoretically is 1, but may be lower for non-ideal conditions due to e.g. particle bounce. It was assumed that the c_max_ value was reached at 120 nm, from where the first decrease in removal efficiency was observed. The c_max_ parameter was determined to 0.53 ± 0.1, which was found as the average removal of particles larger than 120 nm. The fit resulted in a steepness parameter of 2.29 ± 0.8 and a D_50_ value of 73 ± 8 nm, which is close to the theoretical D_50_ value of 55 nm. The determined collection efficiency curve for the MINI impactor is compared to commercially available impactors in the SI.

### Deposition pattern

**Figure 5:**
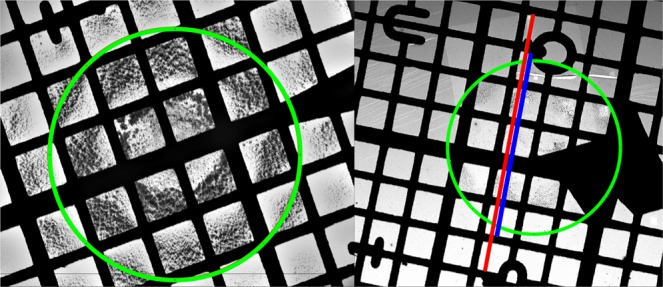
Two examples of impaction spots: Left image depicts an overloaded impaction spot, while the right is sufficiently loaded. Estimated positions of the impactor nozzle are marked in green. The Impaction spot on the right image was investigated further by acquiring two series of images. Imaged areas marked in red and blue.

The PSL aerosol was measured via SMPS in order to obtain a rough estimate of the total number concentration, which was determined to approximately 500.000 cm^−3^. The aerosol was sampled for 10 seconds with the impactor. An additional sample was collected for 10 seconds after diluting the aerosol to a total number concentration of approximately 100.000 cm^−3^. The grids were analyzed by SEM at 10–20 keV and varying magnifications. The grids were found to have localized areas with significantly higher number densities (*μ*m^−2^) than the rest, covering an area of approximately 6 × 6 squares on the TEM grid (approximately 0.1 mm^2^). These areas were concluded to be the parts of the grids situated directly under the impactor orifice during sampling, and will be referred to as impaction spots. The size of the impaction spots were found to fit well with the orifice size, which has a diameter of ca. 0.29 mm. For easy visualization, an example of an overloaded and a sufficiently loaded impaction spot are shown in Fig. 5. The estimated position and size of the impactor orifice are marked with green circles, while two of the imaged areas used for further analysis are marked in red and blue on the right image of Fig. 5.

The particle number densities far from the impact centers were typically an order of magnitude lower than within the impaction spots. However some areas near the very edge of the grids, displayed varying number densities, with some approaching the same level as within the impact areas, while others remained an order of magnitude lower. This indicates that streamlines vary locally near the edge of the grid, making it difficult to define a standard imaging routine in this area, while number densities between the edge and the impact area contain too few particles to allow for a statistical analysis without having to acquire hundreds of images. Furthermore particles outside the impact area were typically sub 100 nm, with only few in the 100–200 nm regime, and none above. It was therefore concluded that the larger particles were almost exclusively collected within the impact area, while particles near the impactor cut-off were collected both within and outside. As a result focus was put on analyzing the impact areas, since the analysis should capture the full range of particle sizes.

**Figure 6:**
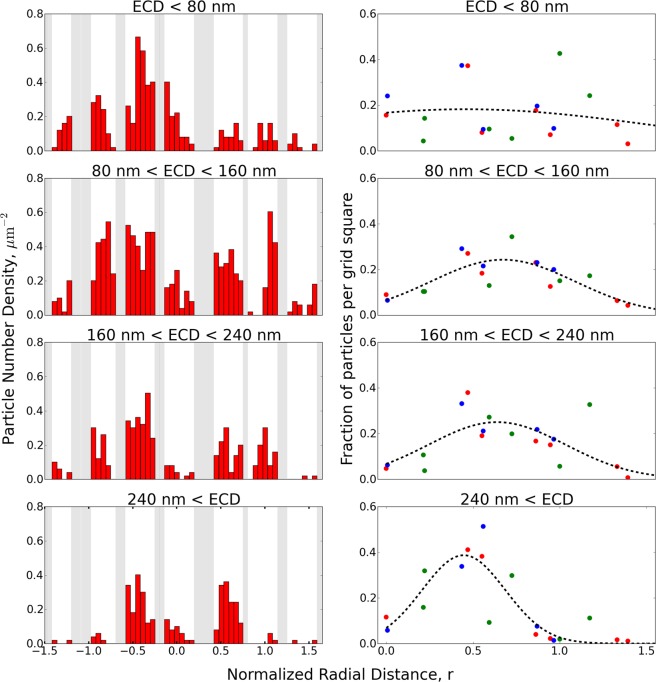
Left side: Particle deposition pattern found from the image sequence marked in red on Fig. 5, visualized as particle number density from each acquired image for each size bin, plotted against the normalized radial distance from the impact center. Grey areas represent discarded images acquired on the Ni grid. Right side: Comparison of the three samples, plotted as fraction of particles from each size bin within each grid square for the three different image data sets plotted against the absolute normalized radial distance from the impact centre. The red and blue data sets are from the areas marked in Fig. 5, while green is from the diluted aerosol. A Gaussian distribution was fitted to the data points (black dashed line) for easy visualization of the impact trend.

Within the impact areas, local particle size distributions were found to vary with distance from the center of impaction. To investigate, a series of images were acquired in a straight line, passing through the center of impaction, covering a distance of up to 1.5 times the orifice radius on either side. The imaging routine was performed once on the grid from the diluted aerosol, while two parallel routines were performed on the undiluted sample, marked in red and blue on Fig. 5. The two parallel measurements were made to determine the variation between PSDs measured from different areas of the same sample, while the diluted sample was included to investigate variations between different samples of the same but diluted aerosol. The routines were all performed at 20 keV and at 20 k magnification with a pixel size of 3.7 nm. This resulted in 39, 27, and 28 images from the red area, blue area, and diluted sample, respectively. The distance from each of the acquired images to the estimated center of impaction was calculated based microscope coordinates. Each image was analyzed using a manually set global or mean adaptive threshold to distinguish particles from substrate. A total of 1195, 938, and 365 particles were recognized from the three image sequences, which were further divided into four size bins based on their equivalent circular diameter (ECD): 0–80, 80–160, 160–240, and >240 nm. The limits of the size bins were chosen to separate the expected PSL sizes of approximately 40, 100, 200, and 500 nm. To visualize the impaction pattern the number density within each image for each of the four size bins were plotted against the normalized radial distance from the impact center in units of orifice radius. The resulting plots for the image sequence marked in red on Fig. 5 are shown in the left side of Fig. 6. Since the STEM detector is unable to produce images when the electron beam is situated on the nickel grid these images were discarded prior to analysis, and are therefore represented as grey areas in the plot. As a result the red bars are arranged in clusters of 5–6 bars, with each cluster representing images within the same grid square. From the plots on the left hand side of Fig. 6 it is seen that particles below 80 nm (top left plot), which include particles close to and below the impactor D_50_ at 73 nm, were found within the entire area underneath the impactor nozzle as well as in the periphery area. The particles were found almost homogeneously distributed within the imaged area, with a slight increase near the impact center. Particles between 80 and 160 nm (second plot on the left), representing particles just above D_50_, were observed within the entire area directly underneath the nozzle, but showed lower number densities in the periphery area. The highest number densities were observed at approximately one orifice radius from the center. Particles with ECD between 160 and 240 nm (third plot on the left) were also observed within the entire area underneath the nozzle, but had much lower densities in the periphery area. Furthermore the highest number densities were found closer to the impact center than for the smaller bins. Particles with ECD larger than 240 nm (bottom left plot), were concentrated in a small area close to the impact center and were rarely found further than half an orifice radius from the impact center.

It is worth noting that the number densities from different locations within the same grid square can vary significantly, which is most likely due to local flow conditions. This effect can be seen in Fig. 5, where the suspended film appears to deflect between the grid bars under the jet pressure, leading to a limited impaction in one side of the squares and an enhanced deposition in the other. To even out this effect the average number of particles for each size bin within each grid square was determined. From this the overall fraction of particles within each grid square for each size bin was calculated, for each of the three data sets. The fractions are plotted against the absolute normalized radial distance, since the impact pattern is expected to be symmetrical around the impact centre. The resulting plots are shown in the right side of Fig. 6, where each color represents a different data set, with the imaged area for the red and blue data points marked in Fig. 5, while the green data set was obtained from the diluted aerosol sample. A Gaussian distribution was fitted to the data points for easy visualization of the impact trend. The particles below 80 nm show a homogeneous deposition trend, indicating deposition is governed by diffusion rather than impaction as expected near the impactor cut-off. Furthermore these particles are on or close to the limit of detection for the microscope. For the three larger size bins clearer deposition peaks are observed, which shifts according to particle size. This clearly illustrates that an increase in particle size, shifts the highest probability of deposition towards the impact center, while narrowing the area in which the particles are impacted. Neglecting particles smaller than the impactor cut-off, the broadest peak is observed for the size bin just above the impactor cut-off, where a deposition fraction close to zero is reached at approximately 1.5 times the orifice radius. It is therefore necessary to include images up to a distance of 1.5 times the orifice radius to capture all particle sizes above the cut-off.

The observed pattern can be compared to computational studies by^37^ and experimental studies by^38^, where the deposition probability was described as a function of distance to the impact center and particle size. Both studies found that particles larger than the impactor D_50_, are governed by their inertia and will deposit mainly in the area directly under the nozzle. Both studies furthermore show that tapered nozzles generate an aerodynamic focusing effect due to the converging flow in the jet, which changes the size of the impacted area as a function of particle size. Particles much larger than D_50_, impact in a localized area smaller than the nozzle close to the impact center, while particles near D_50_ impact within the entire area underneath the nozzle and up to a distance of 1.5 times the orifice radius. The computational study of ^37^ furthermore found that the number density of particles with a given size sharply increase near the edge of their impact area, due to the focusing effect. This trend was however less obvious in the experimental study of ^38^, where only minor increases were observed at the edges. Particles smaller than D_50_ were in both studies found to be governed mainly by the jet streamlines, resulting in a more homogeneous diffusion based deposition within and outside the impacted area, with only a small fraction of the particles being collected.

These findings fit very well with the impaction pattern observed in this study, where particles close to and below D_50_ were observed in the entire imaged area, while particles larger than D_50_ were concentrated underneath the nozzle. Additionally, the same correlation between particle size and impact area was observed, where an increase in particle size result in a decrease in impact area, with the highest number densities found close to the edge of the impacted area rather than near the center. However we did not observe the sharp increase in number density near the very edge of the impact area as described by^37^, but rather found trends similar to those described by^38^.

Based on the observed impaction patterns it was concluded that the PSD observed from individual images was highly dependent on the distance from the impact center. As a result it is necessary to acquire a series of images, representing all distances from the center of impaction to a distance of 1.5 times the orifice radius, in order to include particles from D_50_ and up. Beyond 1.5 times orifice radius, mainly particles near and below D_50_ are deposited with number densities typically an order of magnitude lower than within the impaction spot.

### Comparison to SMPS measurements

The total PSD obtained from EM analysis was found as the average of the PSDs from each of the individual images in the imaging sequence presented in the previous section. The data set used in this section is the imaged area marked in red on Fig. 5 with 1195 particles recognized after segmentation.

**Figure 7:**
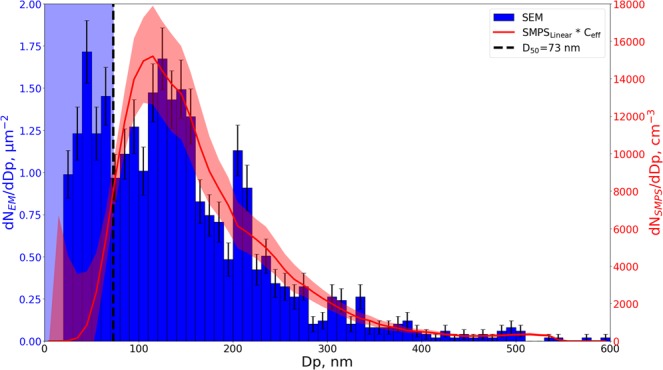
PSD obtained from SEM analysis (blue) plotted with the linear translated and ${{\rm{C}}}_{{\rm{eff}}}^{{\rm{Fit}}}$ adjusted PSD from the SMPS (red). Error bars on the SEM data are given as $\sqrt{{\rm{N}}}$, while the red shaded area indicates the standard deviation of the five SMPS scans and the experimental uncertainty of the ${{\rm{C}}}_{{\rm{eff}}}^{{\rm{Fit}}}$ expression in Fig. 4. The blue shaded area indicates the size detection limit of the SEM, meaning that number concentrations in this area are highly uncertain.

In order to determine if the imaging pattern yielded a representative PSD, a comparison was made to the PSD measured via SMPS, which was found as an average of 5 runs prior to sampling with the impactor. Since spherical latex beads were used with a density of approximately 1, the assumptions of the SMPS hold for the primary particles, allowing direct comparison between the mobility diameter of the SMPS and the ECD from the SEM analysis when taking the impactor collection efficiency into account. This was done by multiplying each of the number concentrations in the SMPS size bins with the corresponding impactor ${{\rm{C}}}_{{\rm{eff}}}^{{\rm{Fit}}}$ for that given particle size determined from the fitted function shown in Fig. 4. Furthermore the size bins of the SMPS were converted from log scale to the linear scale used in the SEM analysis, to allow a direct comparison between the relative shapes of the two PSDs, as plotted in Fig. 7. The SMPS log normal PSD can be found in the SI, along with the linear converted PSD and the ${{\rm{C}}}_{{\rm{eff}}}^{{\rm{Fit}}}$ corrected PSD for comparison. The Error bars on the SMPS PSD in Fig. 7 are found as the standard deviation of the 5 SMPS scans along with the uncertainty of the ${{\rm{C}}}_{{\rm{eff}}}^{{\rm{Fit}}}$ function, found from the experimental data (${{\rm{C}}}_{{\rm{eff}}}^{\exp }$). Error bars of the SEM PSD were found from counting statistics as $\sqrt{N}$.

It is seen that the SEM PSD has clearly distinguishable modes at approximately 50, 140, and 210 nm. The SMPS PSD has a peak at 120 nm with shoulders at 150 and 210 nm, showing reasonable agreement with the 140 and 210 nm peaks of the SEM PSD. Despite the circularity of particles in the 140 and 210 nm size bins (Fig. 10 in the SI), we cannot exclude a possible aggregation of finer particles that could explain some of the differences between the SMPS and SEM particle size distributions. The SEM mode at 50 nm is below the impactor cut-off and is therefore not present in the SMPS PSD, as the number concentration below D_50_ are reduced by the ${{\rm{C}}}_{{\rm{eff}}}^{{\rm{Fit}}}$ correction. Furthermore, the detection limit of the SEM, with the settings used here, is between 20 and 90 nm depending on image quality, and the exact number concentration and size of particles in this range is therefore highly uncertain, as described in the SI. However, the 50 nm peak does show that particles below the impactor cut-off can be collected on the lowest stage of the MINI impactor, but it is not possible to relate their number concentration to that of the larger sizes. If sub D_50_ particles are of interest it is recommended to investigate the sample using TEM rather than SEM to overcome the resolution issues. It should be noted that the limit of detection and size uncertainty will be lower for particles consisting of higher Z elements than carbon, as they provide a better contrast on the SEM images. Particles close to 100 nm are slightly underestimated in the SEM PSD compared to the SMPS PSD. This can be partly explained by the uncertainties in the determined D_50_ and collection efficiency expression, where a small shift to a larger D_50_ could explain why fewer 100 nm particles are found within the impact area, which could be the case if D_50_ is dependent on bounce effects. Alternatively the difference may arise from particles depositing outside the impacted area as they are close to the impactor D_50_, making them under represented within the imaged area. This could furthermore explain why the ratio between the 210 and 140 nm peaks is slightly higher for the SEM PSD compared to the SMPS.

In order to investigate the reproducibility and uncertainty using the proposed image sequence, a comparison was made between the PSDs resulting from the two parallel image sequences (marked in red and blue in Fig. 5). An additional comparison was made between two perpendicular imaging routines, acquired from the diluted aerosol sample, giving a total of four different image sequences. The resulting PSDs are shown in the SI, along with the SMPS measurements for comparison. In general a good agreement was found between the shapes of the PSDs for particles larger than D_50_. All four PSDs displayed the main modes at approximately 140 and 210 nm with only minor shifts, which were most likely caused by uncertainties from the image quality or the manual image segmentation process. Furthermore the ratios between the two peaks were similar for all four samples. However, for particles close to or below D_50_, a significant variation was observed between the samples, again related to the limit of detection for the microscope. A mode below D_50_ was however evident in all SEM PSDs, but it shifted up to 30 nm between samples. Above the impactor cut-off the imaging routine was able to give reproducible PSDs both for data sets acquired from the same sample and from different samples of the same aerosol before and after dilution.

The total number concentration of the sampled aerosol is highly relevant for cases where most online measurement techniques are known to struggle e.g. in cases with fibers or complex mixed aerosols with varying composition and density. Based on the agreement with SMPS, the number concentration can be estimated from SEM for particles larger than D50 as the uncertainty below that size becomes to large. In order to link the measured impactor number densities (N_*Imp*,*dp*_) to the measured total aerosol concentrations (C_SMPS_) the following equation was used:

2 $$tQ{C}_{{\rm{SMPS}}}=A\,\sum _{dp=73}^{\infty }\,\frac{{N}_{{Imp},dp}}{{C}_{eff,dp}^{Fit}}$$

where *t* is the sampling time, *Q* is the flow rate through the impactor, ${{\rm{C}}}_{eff,dp}^{Fit}$ is the impactor collection efficiency for size *dp*, and *A* is assumed to be a constant to be determined. Apart from A, all parameters in Equation 2 are known for the four image sequences described earlier. The *A* parameter was found to be $1.12\ast {10}^{6}\pm 0.60\ast {10}^{6}$, showing that the estimate has an uncertainty of approximately 50% for these samples. In cases where aerosols consist of particles rather similar in composition and shape, it would therefore be possible to estimate a total particle number concentration from impactor samples alone. Impaction can then be used as a stand alone method if a series of samples are collected with varying sampling times to achieve a range of coverages. It should however be noted that the *A* parameter may be affected by bounce effects or if larger/smaller particles are investigated than here.

### Sampling time

**Figure 8:**
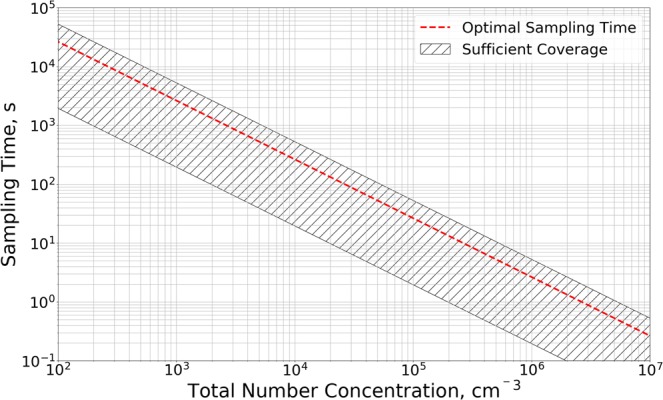
Plot of $t={S}_{p}$/$(QC)$, where the determined upper and lower limits for number of sampled particles (*S*_*p*_) has been inserted. The plot links the total number concentration (*Q*) measured by e.g. SMPS to the sampling time (*t*), ensuring sufficient sample coverage. The hashed area is within the upper and lower limits, while the red dashed line marks the optimal sampling time.

A series of samples were collected with the impactor to estimate the optimal sampling time, since the particle loading on a TEM grid is essential for its use in EM analysis. Three aerosols were generated with the setup described previously; yielding total SMPS number concentrations of approximately 10.000, 100.000, and 500.000 cm^−3^, which were dominated by particles between 100 and 200 nm. Each of the three aerosols were sampled 3–4 times with the impactor, collecting between 5 and 60 seconds, yielding a total of 10 samples, displaying a broad range of particle coverages on the grids. To assess if the samples were overloaded they were analyzed by SEM, where the highest populated areas were assessed manually and imaged. These areas were typically at a distance of 100 *μ*m from the impact center, corresponding to a distance of 0.7 times the orifice radius. The particle loading was assessed by determining the percentage of the imaged area recognized as particles. Examples of segmented images are shown in the SI, along with a table containing the total number concentration measured by SMPS, the sampling times, and the determined coverages. The total number of particles going through the impactor (*S*_*p*_) during each sampling was calculated using: ${S}_{p}=tQ{C}_{SMPS}$. The impactor C_eff_ was not used here. Based on the observations it was concluded that a higher than optimal coverage would lead to significant agglomerate formation from co-depositing particles on top of each other. It was furthermore concluded that a lower boundary of particle coverage also exists where a meaningful statistical analysis is no longer possible. The upper and lower limits for *S*_*p*_ were determined to be 6.7$6.7\ast {10}^{7}$ and $3.9\ast {10}^{6}$ particles, as samples within this range contained sufficient particles for analysis, without significant co-deposition. Based on these limits and by rearranging the equation to $t={S}_{p}$/$(Q{C}_{SMPS})$, the total particle number concentration can be linked to sampling time. The expressions are plotted in Fig. 8, which can be used to read the optimal sampling time based on a total particle number concentration measured by e.g. SMPS or CPC. The midpoint between the two sampling limits is marked with a red dashed line, and should be interpreted as the optimal sampling time.

Before using this graph to determine the optimal sampling time, it should be noted that it was determined from a PSD dominated by 100–200 nm particles, without taking into account the impactor collection efficiency. The optimal sampling time may therefore vary with other impactors or if other particle sizes are dominating. Furthermore the optimal sampling time may change depending on the collection efficiency of the particles, where a higher sticking coefficient (less bounce) would need shorter sampling times. It is therefore recommended to collect several samples both with higher and lower sampling time than the optimal found from Fig. 8, thereby obtaining TEM grids with a broad range of particle number densities.

### Suitability for risk assessment

The developed method has unique features that will be valuable for the required characterization of aerosol particle populations. In terms of use, the impactor is small and easy to transport, making it possible to collect samples with minimal equipment. Sampling times are short, typically below a minute, allowing for sampling of peak emissions. The TEM grids in the impactor are easy to exchange and store, allowing for several samples to be collected during a field measurement. It can be advantageous to have an estimate of aerosol particle concentration prior to impactor collection, as it makes it possible to estimate a sampling time for optimal coverage on the TEM grids. Samples can also be collected without such an estimate, but then it is recommended to vary the sampling time significantly (e.g. 1,10,100 sec) to ensure a broad range of particle coverages, allowing total number of particles to be estimated based on calibrated *A* factors for the sampling system. An automated image acquisition routine, using the proposed image sequence, can analyze a sample in 30–180 minutes, depending on the particle coverage. The analysis will give up to 40 images at 20 k magnification, with a detection limit between 50 and 100 nm, as well as the elemental composition of each individual particle from the automated EDS analysis performed on all particles located by the segmentation procedure. Automated EDS analysis is not presented in detail in this work, as the elemental composition of the carbon based particles would not be distinguishable from the carbon based Formvar substrate. Some preliminary results from an ongoing study are presented in the SI as an example of automated EDS analysis. Alternatively, many examples of automated EDS analysis can be found in the literature^49–52^.

Careful visual inspection should be performed to ensure the segmentation is trustworthy before extracting physical parameters for individual particles including area, ECD, circularity, and aspect ratio etc. Two different examples of circularity and aspect ratio distributions for the particles detected from the image sequence marked in red on Fig. 5 are presented in the SI. Here high circularities close to one and low aspect ratios are found for the 210 nm peak, indicating spherical particles, while the less clear 120–150 nm peak, indicate that this peak could contain a significant number of agglomerates. The circularity between the two peaks drop, while the aspect ratio increases, which suggests that intermediate sizes of the aerosolized particles are dominated by agglomerates rather than single spheres. This becomes even more obvious in the SI plot, where PSDs are plotted for particles fulfilling specified shape criterias e.g. only high circularity particles (circularity > 0.90).

The fiber paradigm is of high importance in toxicological assessments, but existing online aerosol analysis instruments are known to have difficulties with measurements of fiber samples. Fibers’ pathway through the impactor system must be as particles of sizes somewhere between their smallest and largest dimension. The method will likely perform equally well for particles and fibers with dimensions in the tested range, given the uniform weighting of the different particle sizes from the images when taken across 1.5 times the impactor orifice diameter as discussed with Fig. 6. For fibers a lower deposition density will likely be preferable to avoid co-deposition.

## Conclusion

This work has provided a reproducible quantification procedure for collecting, imaging, and analyzing aerosol populations using impaction onto TEM grids and automated SEM analysis. Transmission mode SEM images gave better contrast than secondary or backscatter detection for the carbon (low Z) dominated particles. A clear particle size dependent impact pattern was observed (Fig. 6), similar to what has been reported in the literature. Based on the observed pattern a simple and reproducible imaging routine was established, in order to ensure that all particle sizes were represented by the analysis. The PSD resulting from the imaging routine was compared to SMPS measurements (Fig. 7), showing good agreement between PSD shapes for particles with ECD larger than the impactor cut off diameter D_50_. The procedure can determine shape distributions for the sampled aerosol, as shown in the SI, enabling a highly detailed individual particle analysis capable of distinguishing particle types e.g. fibers, agglomerates, and single particles if the recommendations to avoid co deposition of particles is followed (Fig. 8). The method can provide an estimate of the total particle number concentration of the sampled aerosol using the determined parameter *A* and Equation 2. The method is off-line and can be used alone but should preferably be coupled with fast response instruments to provide real-time data for assessing concentration fluctuations and to determine when, where, and possibly how long to collect samples. If coupled with EDS, elemental composition distributions can also be measured, to distinguish harmless particles e.g. sea salt or crustal dust from hazardous particles e.g. transition metals or soot, as exemplified in the SI.

The method is therefore capable of giving a detailed aerosol characterization beyond what is achievable by the more common real-time instruments such as SMPS, ELPI, CPC, diffusion chargers, or FMPS, by providing both size, shape, and composition distributions of an aerosol population in time windows of a few minutes, and is expected to allow a reasonable fiber assessment as well (currently under investigation). The more precise statistical data on physical and elemental composition achievable by the proposed method will make it possible to distinguish different types of particles and their relative contribution to the aerosol population^53,54^, and hence provide an improved foundation for risk assessments, exposure assessments for epidemiological studies, and the development of preventive strategies for e.g. aerosol pollution.

## Supplementary information

Supplementary Information

## Data Availability

The datasets and codes used and/or analysed during the current study are available from the corresponding author on reasonable request.

## References

[1] Landrigan Philip J, Fuller Richard, Acosta Nereus J R, Adeyi Olusoji, Arnold Robert, Basu Niladri (Nil), Baldé Abdoulaye Bibi, Bertollini Roberto, Bose-O'Reilly Stephan, Boufford Jo Ivey, Breysse Patrick N, Chiles Thomas, Mahidol Chulabhorn, Coll-Seck Awa M, Cropper Maureen L, Fobil Julius, Fuster Valentin, Greenstone Michael, Haines Andy, Hanrahan David, Hunter David, Khare Mukesh, Krupnick Alan, Lanphear Bruce, Lohani Bindu, Martin Keith, Mathiasen Karen V, McTeer Maureen A, Murray Christopher J L, Ndahimananjara Johanita D, Perera Frederica, Potočnik Janez, Preker Alexander S, Ramesh Jairam, Rockström Johan, Salinas Carlos, Samson Leona D, Sandilya Karti, Sly Peter D, Smith Kirk R, Steiner Achim, Stewart Richard B, Suk William A, van Schayck Onno C P, Yadama Gautam N, Yumkella Kandeh, Zhong Ma (2018). The Lancet Commission on pollution and health. The Lancet.

[2] Uherek TH (2010). Transport impacts on atmosphere and climate: Land transport. Atmospheric Environment.

[3] Vu TV (2017). Physical properties and lung deposition of particles emitted from five major indoor sources. Air Quality, Atmosphere and Health.

[4] Viitanen AK, Uuksulainen S, Koivisto AJ, Hämeri K, Kauppinen T (2017). Workplace measurements of ultrafine particles-A literature review. Annals of Work Exposures and Health.

[5] Koponen IK, Koivisto AJ, Jensen KA (2015). Worker exposure and high time-resolution analyses of process-related submicrometre particle concentrations at mixing stations in two paint factories. Annals of Occupational Hygiene.

[6] Kaur I, Agrawal R (2007). Nanotechnology: A New Paradigm in Cosmeceuticals. Recent Patents on Drug Delivery & Formulation.

[7] Maynard AD (2007). Nanotechnology: The next big thing, or much ado about nothing?. Annals of Occupational Hygiene.

[8] Sadeghi R, Rodriguez RJ, Yao Y, Kokini JL (2017). Advances in Nanotechnology as They Pertain to Food and Agriculture: Benefits and Risks. Annual Review of Food Science and Technology.

[9] Som C (2010). The importance of life cycle concepts for the development of safe nanoproducts. Toxicology.

[10] Handy RD, Shaw BJ (2007). Toxic effects of nanoparticles and nanomaterials: Implications for public health, risk assessment and the public perception of nanotechnology. Health, Risk and Society.

[11] Carvalho T, Peters J, Williams R (2011). Influence of particle size on regional lung deposition - what evidence is there?. Int J Pharm.

[12] Donaldson K, Poland CA (2013). Nanotoxicity: Challenging the myth of nano-specific toxicity. Current Opinion in Biotechnology.

[13] Cassee FR, Héroux ME, Gerlofs-Nijland ME, Kelly FJ (2013). Particulate matter beyond mass: Recent health evidence on the role of fractions, chemical constituents and sources of emission. Inhalation Toxicology.

[14] Oberdörster Günter, Oberdörster Eva, Oberdörster Jan (2005). Nanotoxicology: An Emerging Discipline Evolving from Studies of Ultrafine Particles. Environmental Health Perspectives.

[15] Saber AT (2014). Particle-induced pulmonary acute phase response may be the causal link between particle inhalation and cardiovascular disease. Wiley Interdisciplinary Reviews: Nanomedicine and Nanobiotechnology.

[16] Boulanger G (2014). Quantification of short and long asbestos fibers to assess asbestos exposure: A review of fiber size toxicity. Environmental Health: A Global Access Science Source.

[17] Braakhuis Hedwig M, Park Margriet VDZ, Gosens Ilse, De Jong Wim H, Cassee Flemming R (2014). Physicochemical characteristics of nanomaterials that affect pulmonary inflammation. Particle and Fibre Toxicology.

[18] McMurry PH (2002). A review of atmospheric aerosol measurements. Developments in Environmental Science.

[19] Kuhlbusch TA, Asbach C, Fissan H, Göhler D, Stintz M (2011). Nanoparticle exposure at nanotechnology workplaces: A review. Particle and Fibre Toxicology.

[20] El Yamani M (2012). Revision of french occupational exposure limits of asbestos and recommendation of measurement method: Can the dimensional characteristics of the asbestos fibers (long, thin, short) be taken into account?. Critical Reviews in Environmental Science and Technology.

[21] Eypert-Blaison C, Romero-Hariot A, Clerc F, Vincent R (2018). Assessment of occupational exposure to asbestos fibers: Contribution of analytical transmission electron microscopy analysis and comparison with phase-contrast microscopy. Journal of Occupational and Environmental Hygiene.

[22] Newbury DE, Ritchie NWM (2013). Is scanning electron microscopy/energy dispersive X-ray spectrometry (SEM/EDS) quantitative?. Scanning.

[23] Laskin A, Cowin JP (2001). Automated single-particle SEM/EDX analysis of submicrometer particles down to 0.1 um. Analytical Chemistry.

[24] Arndt J (2016). Scanning electron microscopy-energy dispersive X-ray spectrometry (SEM-EDX) and aerosol time-of-flight mass spectrometry (ATOFMS) single particle analysis of metallurgy plant emissions. Environmental Pollution.

[25] Margiotta S, Lettino A, Speranza A, Summa V (2015). PM1geochemical and mineralogical characterization using SEM-EDX to identify particle origin - Agri Valley pilot area (Basilicata, southern Italy). Natural Hazards and Earth System Sciences.

[26] Hodoroaba VD (2016). Characterisation of nanoparticles by means of high-resolution SEM/EDS in transmission mode. IOP Conference Series: Materials Science and Engineering.

[27] Thomassen Y (2006). Ultrafine particles at workplaces of a primary aluminium smelter. Journal of environmental monitoring: JEM.

[28] Koivisto AJ (2018). Occupational exposure during handling and loading of halloysite nanotubes – A case study of counting nanofibers. NanoImpact.

[29] Lieke KI (2013). Micro- and nanostructural characteristics of particles before and after an exhaust gas recirculation system scrubber. Aerosol Science and Technology.

[30] Maskey S (2010). The influence of collecting substrates on the single-particle characterization of real atmospheric aerosols. Analytica Chimica Acta.

[31] Casuccio GS (2004). Measurement of fine particulate matter using electron microscopy techniques. Fuel Processing Technology.

[32] Jones T, Moreno T, BéruBé K, Richards R (2006). The physicochemical characterisation of microscopic airborne particles in south Wales: A review of the locations and methodologies. Science of the Total Environment.

[33] Dudkiewicz A (2011). Characterization of nanomaterials in food by electron microscopy. TrAC - Trends in Analytical Chemistry.

[34] Sielicki P, Janik H, Guzman A, Namieśnik J (2011). The progress in electron microscopy studies of particulate matters to be used as a standard monitoring method for air dust pollution. Critical Reviews in Analytical Chemistry.

[35] Capannelli G, Castello E, Comite A, Costa C, Mamolini G (2011). Electron microscopy characterization of airborne micro- and nanoparticulate matter. Journal of Electron Microscopy.

[36] Kandler, K. An aerosol sampler for single particle analysis. In *Paper Presented at European Aerosol Conference 2009* (2009).

[37] Feng J. Q. (2017). A Computational Study of Particle Deposition Patterns from a Circular Laminar Jet. Journal of Applied Fluid Mechanics.

[38] Sethi V, John W (1993). Particle impaction patterns from a circular jet. Aerosol Science and Technology.

[39] Marple VA, Willeke K (1976). Impactor design. Atmospheric Environment (1967).

[40] Newton GJ, Raabe OG, Mokler BV (1977). Cscade Impactor Design and Performance. Journal of Aerosol Science.

[41] Raabe OG, Braaten DA, Axelbaum RL, Teague SV, Cahill TA (1988). Calibration studies of the drum impactor. Journal of Aerosol Science.

[42] Marple VA, Liu BY (1974). Characteristics of Laminar Jet Impactors. Environmental Science and Technology.

[43] Bradski, G. The OpenCV Library. *Dr Dobbs Journal of Software Tools* **25**, 120–125, 10.1111/0023-8333.50.s1.10, 1308.2414 (2000).

[44] Asbach C (2016). Silicone sampling tubes can cause drastic artifacts in measurements with aerosol instrumentation based on unipolar diffusion charging. Aerosol Science and Technology.

[45] Knutson EO, Whitby KT (1975). Aerosol classification by electric mobility: apparatus, theory, and applications. Journal of Aerosol Science.

[46] Bateman AP, Belassein H, Martin ST (2014). Impactor apparatus for the study of particle rebound: Relative humidity and capillary forces. Aerosol Science and Technology.

[47] Seung JL, Demokritou P, Koutrakis P (2005). Performance evaluation of commonly used impaction substrates under various loading conditions. Journal of Aerosol Science.

[48] Winklmayr W, Wang HC, John W (1990). Adaptation of the twomey algorithm to the inversion of cascade impactor data. Aerosol Science and Technology.

[49] Xie RK, Seip HM, Liu L, Zhang DS (2009). Characterization of individual airborne particles in Taiyuan City, China. Air Quality, Atmosphere and Health.

[50] Fan J (2016). Classification and chemical compositions of individual particles at an eastern marginal site of Tibetan Plateau. Atmospheric Pollution Research.

[51] Roy D, Singh G, Gosai N (2015). Identification of possible sources of atmospheric PM10 using particle size, SEM-EDS and XRD analysis, Jharia Coalfield Dhanbad, India. Environmental Monitoring and Assessment.

[52] Pietrodangelo A, Pareti S, Perrino C (2014). Improved identification of transition metals in airborne aerosols by SEM-EDX combined backscattered and secondary electron microanalysis. Environmental Science and Pollution Research.

[53] Durdziński Paweł T., Dunant Cyrille F., Haha Mohsen Ben, Scrivener Karen L. (2015). A new quantification method based on SEM-EDS to assess fly ash composition and study the reaction of its individual components in hydrating cement paste. Cement and Concrete Research.

[54] Mishra SK (2017). Morphology, Mineralogy and Mixing of Individual Atmospheric Particles Over Kanpur (IGP): Relevance of Homogeneous Equivalent Sphere Approximation in Radiative Models. Mapan-journal of Metrology Society of India.

