# Supplementary material for: Improving the foundation for particulate matter risk assessment by individual nanoparticle statistics from electron microscopy analysis
Source: Sci Rep. 2019 May 30;9:8093. doi: 10.1038/s41598-019-44495-7 (PMC6542787; doi:10.1038/s41598-019-44495-7)
Supplement: Supplementary file 1 — Supplementary Information [file 41598_2019_44495_MOESM1_ESM.pdf]

Improving the foundation for particulate matter risk assessment by individual nanoparticle statistics from electron microscopy analysis

Anders Brostrøm[1,3,*], Kirsten Inga Kling[1], Ismo Kalevi Koponen[2], Karin Sørig Hougaard[3], Konrad Kandler[4], and Kristian Mølhave[1]

[1]National Centre for Nano Fabrication and Characterization (DTU Nanolab), Technical University of Denmark, 2800 Kgs. Lyngby, Denmark
[2]Metrology and Air Environment, Force Technology, 2605 Brøndbyvester, Denmark
[3]National Research Centre for the Working Environment, 2100 Copenhagen, Denmark
[4]Institut für Angewandte Geowissenschaften, Technical University of Darmstadt, 64287 Darmstadt, Germany
[*]abbl@dtu.dk

ABSTRACT

Supporting Information to the manuscript titled: Improving the foundation for particulate matter risk assessment by individual nanoparticle statistics from electron microscopy analysis

Impactor Design Parameters

The design parameters used in the fabrication of the MINI impactor are specified in Table 1, while the abbreviations are indicated in the cross sectional view shown in Fig. 1.

Criteria	value
s/w	1.3
l/w	1 for w > 0.5 mm, else 0.5 mm
w_s	3 for w > 0.5 mm, else 1.5 mm
α	45°
D_t	10.0 mm
D_s	9.95 mm
D_f	7.00 mm
D_c	4.00 mm
f_d	0.90 mm
f_h	1.20 mm
O-rang spec.	8 mm x 1 mm

Table 1. Dimension used in the design of the MINI impactor. Abbreviations are specified in Figure 1.

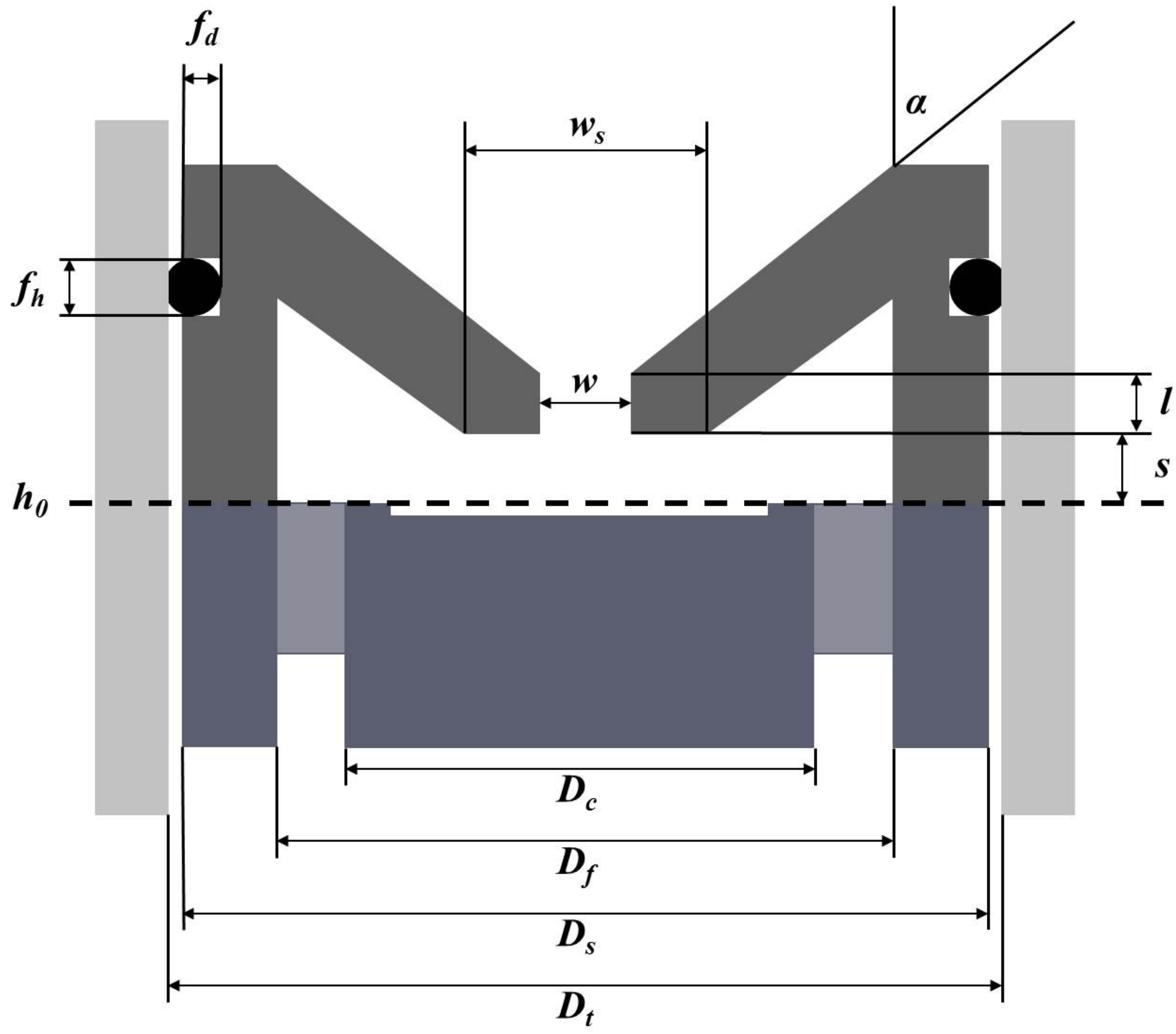

Figure 1. Cross section view of a MINI impactor stage and jet. Measures of the marked dimensions are provided in Table 1.

Image segmentation and detection limit of SEM

Choice of segmentation method was based on the quality of the treated image, where the best results for noisy images were found with the adaptive technique, while a simple global threshold was sufficient for high contrast images. Recognized particles touching the image borders were discarded, as well as particles with equivalent circular diameters (ECD) smaller than 20 nm (5 pixels). The size criteria was chosen to minimize the number of miss classified substrate artefacts, which often occurred on noisy images, where the 40 nm low contrast latex particles were difficult to distinguish on the Formvar film. However, the actual size limitation of particle detection and uncertainty in measured particle size are difficult to assess as they depend on the thickness and elemental composition of both substrate and particle as well as microscope settings such as acceleration voltage and magnification. To further complicate the issue, focus and astigmatism may change between individual images, resulting in additional uncertainty. Many techniques currently exist for assessing particle size uncertainty and limit of detection from EM images[1,2], but no standard methodology currently exists.

In this work the limit of detection was assessed by analyzing the intensity histograms of the acquired images, fitting a Gaussian distribution to the intensity peaks, corresponding to the substrate pixels. This gave a mean intensity for the substrate pixels in each image along with a substrate intensity standard deviation (σ), describing the level of noise in the given image. The σ value varied between intensities of 1 and 30, depending on the quality of the image. It was assumed that particles could be distinguished from the substrate, once the pixel intensity reached values deviating 3σ from the substrate mean, as this corresponds to the base of the Gaussian histogram peak, as seen in the top right plots of Figures. 2 and 3. The number of pixels needed to reach this deviation was found to be both particle size and image quality dependant. For low noise images, the sigma value was close to 1 and the number of pixels needed to deviate from the mean by 3σ was 2-4 pixels for sub 100 nm particles. As a result the smallest detectable particles on a low noise image was 4-8 pixels, including both sides of the particle, which corresponds to roughly 15-30 nm. For noisy images this limit increased by a factor of three, leaving the smallest detectable particles between 45 and 90 nm. Examples of a low noise and noisy image are shown in the top left of Figures 2 and 3, while their Gaussian fitted histograms are shown in the top right. Examples of profile plots for a large and small particle from both the noisy and low noise images are shown in the bottom of Figures 2 and 3, where the transition from substrate mean intensity to mean minus 3σ has been marked. It should be noted that these particle detection limits were assessed for low contrast latex beads, and will be much lower for higher contrast particles. However, samples may need to be analyzed by TEM rather than SEM, if the majority of the particles are below 50 nm and consist of low Z elements with a weak contrast.

For particles larger than 200 nm, the number of pixels needed to reach the three sigma deviation was smaller, typically 1-2 pixels for low noise images and 3-7 pixels for noisy images. These values can be used as indicators of sizing uncertainties, which therefore vary from 7-50 nm, depending on the image quality.

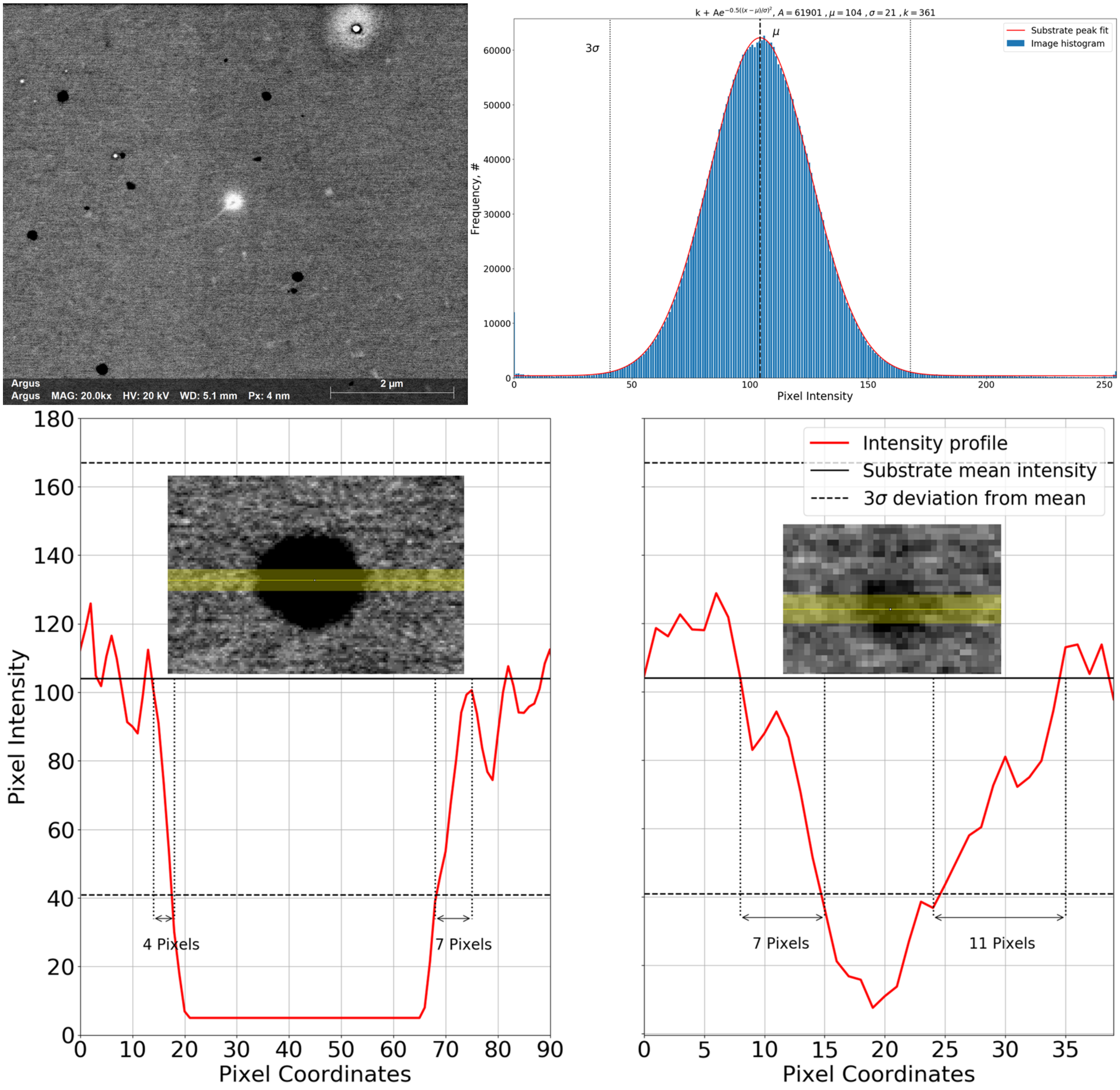

Figure 2. Top Left: Example of a noisy STEM image. Top Right: Histogram of the noisy image, where the substrate peak has been fitted with a Gaussian function to determine the mean and σ values. Bottom: A profile plot of the pixel intensities going across a large and a small particle from the noisy image. The number of pixels needed to go from mean substrate intensity below the 3σ deviation is marked.

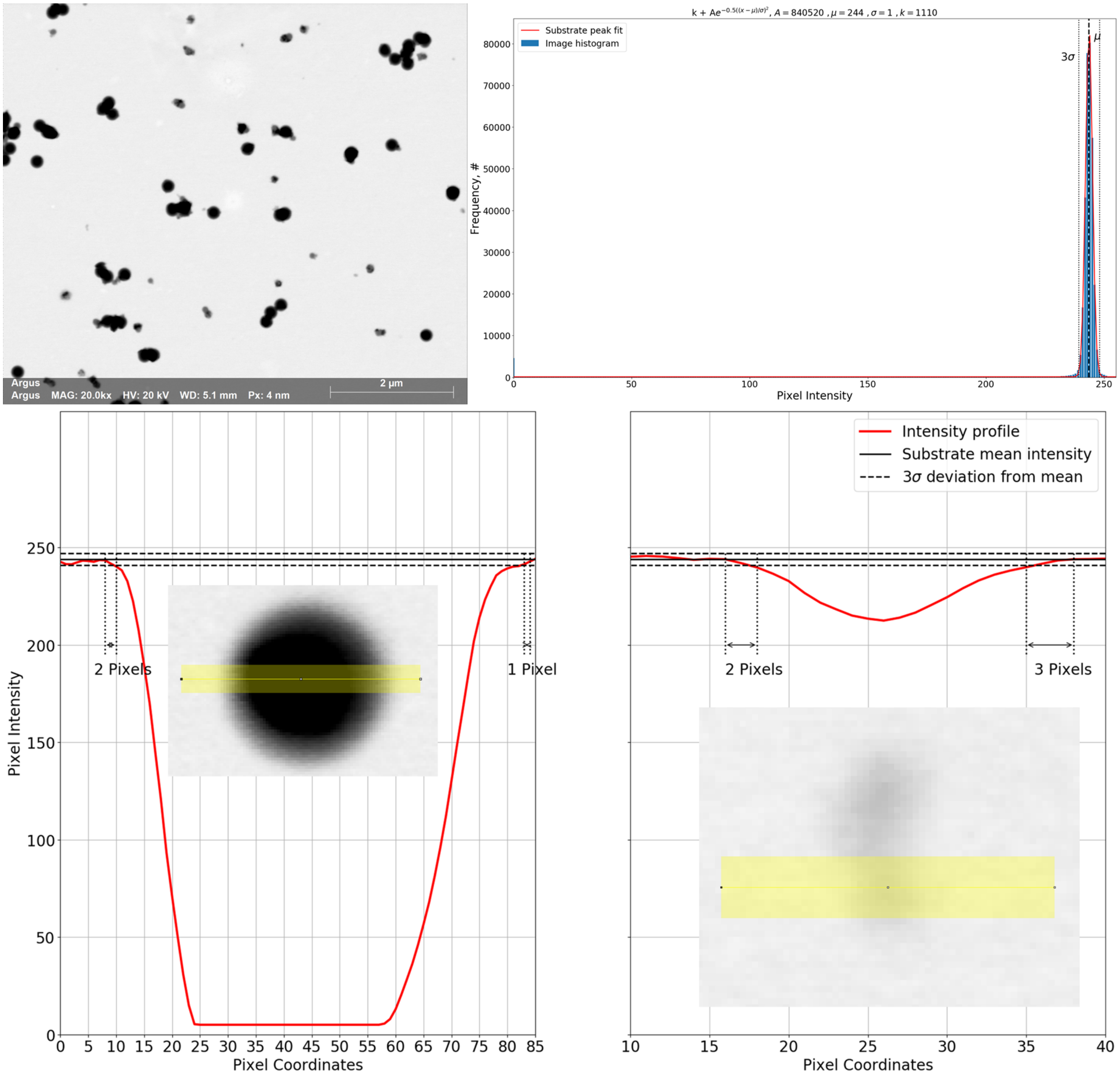

Figure 3. Top Left: Example of a low noise STEM image. Top Right: Histogram of the low noise image, where the substrate peak has been fitted with a Gaussian function to determine the mean and σ values. Bottom: A profile plot of the pixel intensities going across a large and a small particle from the low noise image. The number of pixels needed to go from mean substrate intensity below the 3σ deviation is marked.

CPC Data for Determining Impactor Collection Efficiency

The monodisperse size distributions selected by DMA and detected by CPCs before and after the MINI impactor.

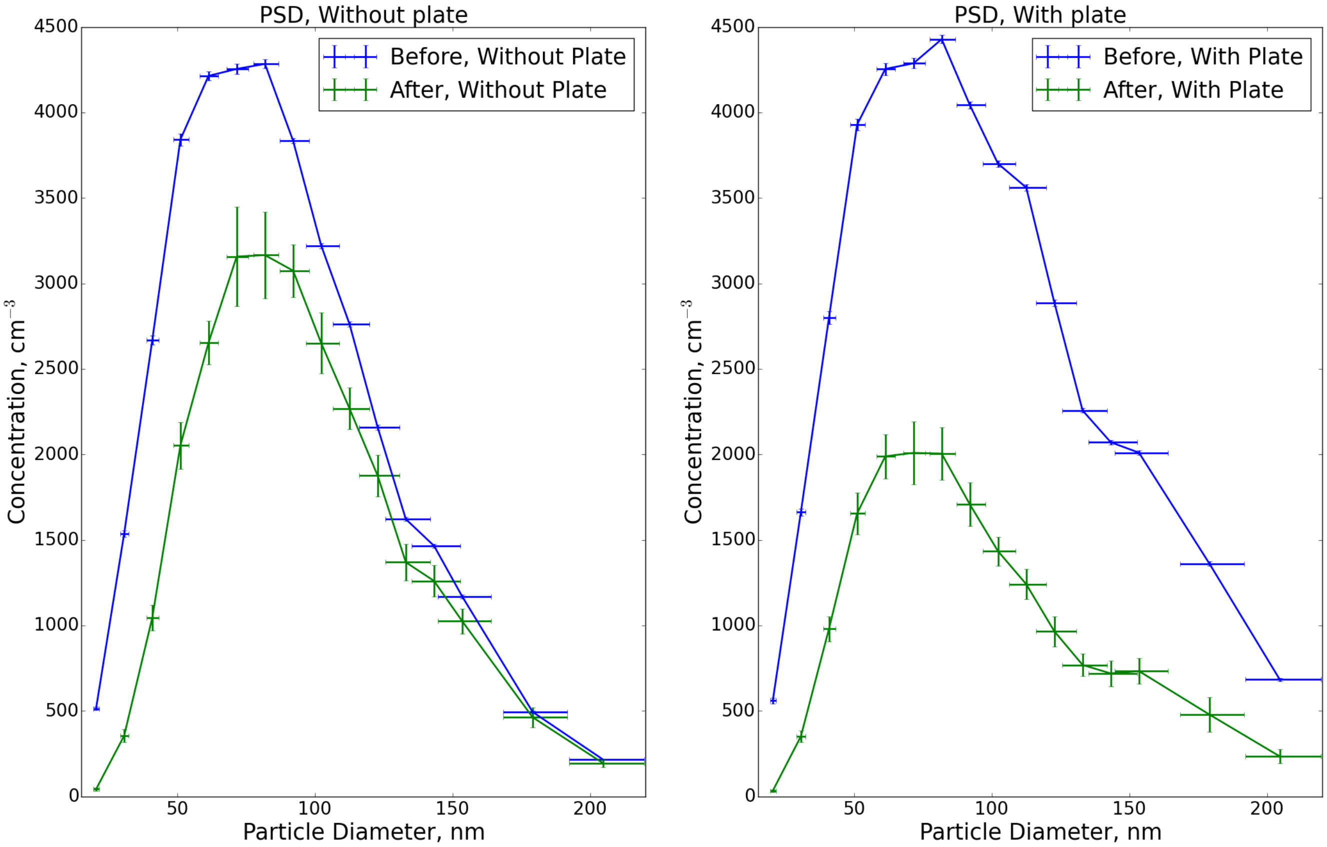

Figure 4. PSDs generated from atomizing a solution of 100 ml nanopure water with 2 drops of 42 $\pm$ 0.5 nm, 75.8 $\pm$ 1, 102.7 $\pm$ 1.3, 150 $\pm$ 1.9, and 207 $\pm$ 2.6 nm 1 wt% PSL solutions. Particle sizes were selected with a DMA and measured with two CPCs, one before and one after the impactor, which was installed with a 0.29 mm orifice either with or without an impaction plate underneath. A schematic of the setup used, is shown in the main text.

Comparing MINI to Commercial Impactors

If it is assumed that the D_{50} and steepness parameter are unaffected by bounce, the c_{max} value in Equation found in the main text can be changed to one. This allows a direct comparison to more well characterized and commercially available impactors, as shown in Figure 5, where the C_{eff} curves of the second to last stage of the ELPI and the last stage of the Berner impactor are plotted for comparison[3,4].

It is seen that the sharpness of the MINI impactor C_{eff} curve is slightly lower than for the commercially available impactors, however the D_{50} are very close with values of 54, 63, and 73 nm for the ELPI, Berner, and MINI impactor respectively.

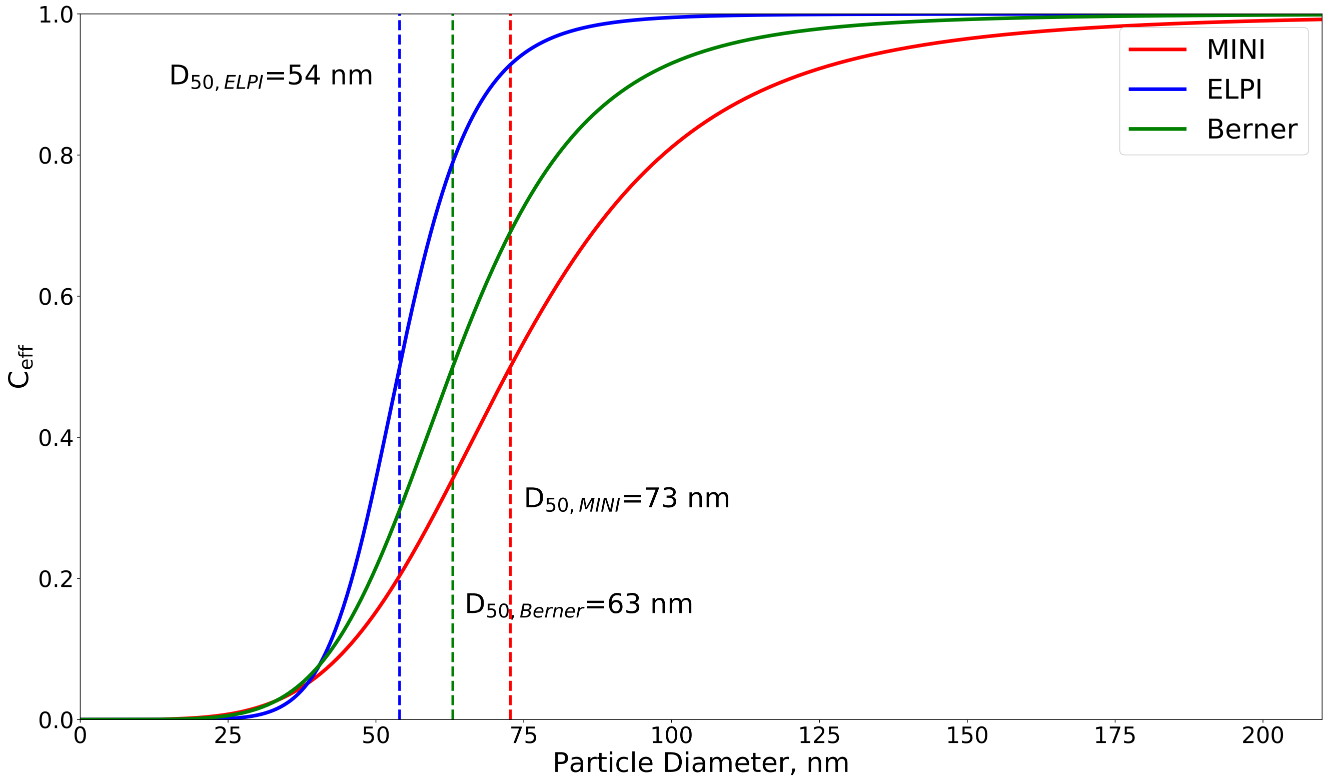

Figure 5. Comparison of C_{eff}^{Fit} curves for the last stage of the MINI impactor, second to last stage of the ELPI, and last stage of the Berner impactor.

Conversion of SMPS PSD and PSD Comparison

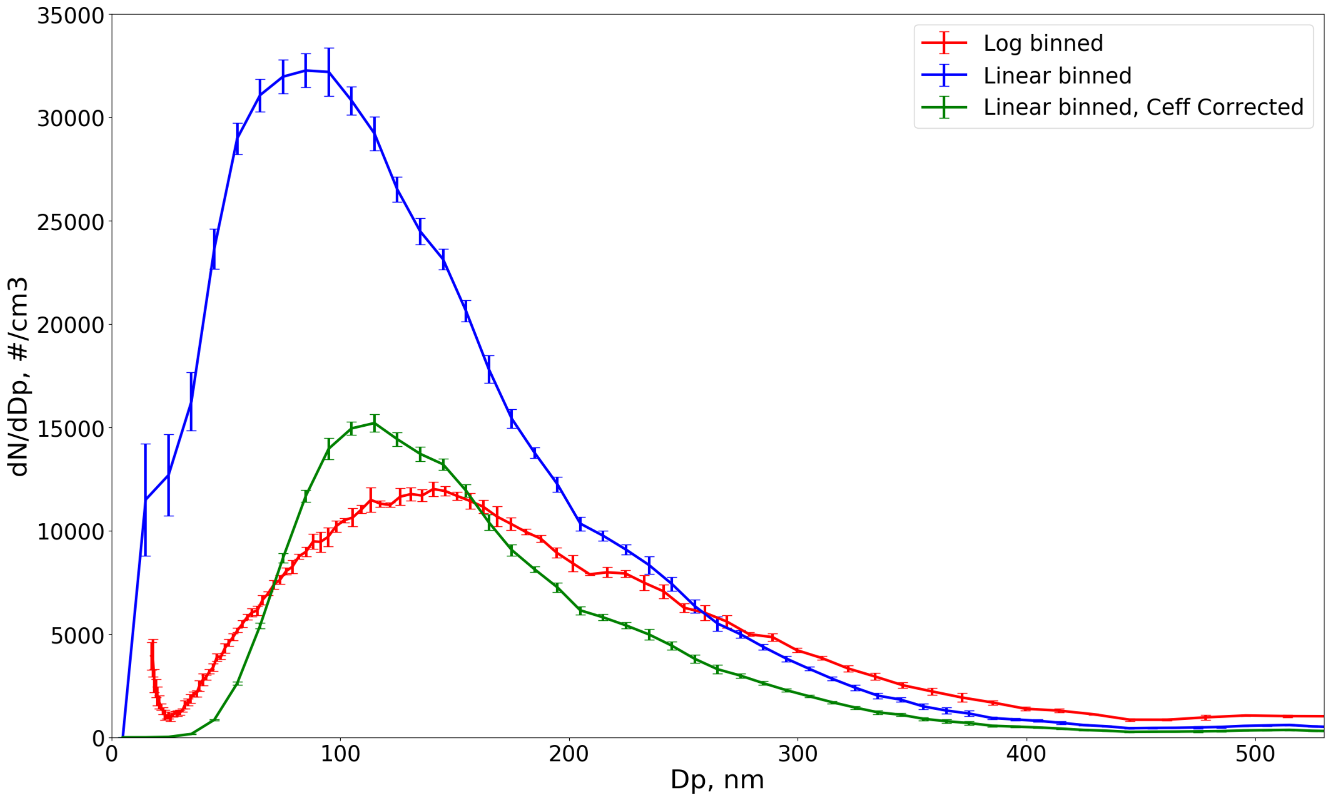

Figure 6. PSDs obtained as an average of five SMPS runs prior to sampling with the impactor. The error bars are determined as the standard deviation between the five runs. The size bins of the red PSD are log normal and plotted directly from the SMPS data. The blue PSD has been converted from the log scale to a linear scale, using the size bin limits of the TSI instrument manual and the SMPS transfer function. The green PSD has been corrected for C_{eff} using the fitted expression found in the main text. Here the C_{eff} uncertainty was not included in the error bars.

PSDs determined from analyzing impacted PSL aerosols, using the linear imaging routine are presented in blue in Fig. 7. The average of five SMPS runs, which were converted from log scale to linear scale, and which were measured 2 minutes before to 2 minutes after the EM sampling are presented in red for comparison. Errors on the EM data is determined from counting statistics as 1/sqrt(N). The SMPS errors were found as standard deviations from the five SMPS runs, as well as from the uncertainties in the collection efficiency expression in the main text. The top PSD in Fig. 7 is from the impactor sample marked in blue in the Figure found in the main text, while the middle and bottom PSDs in Fig. 7 are from the same aerosol sample, using perpendicular imaging routines.

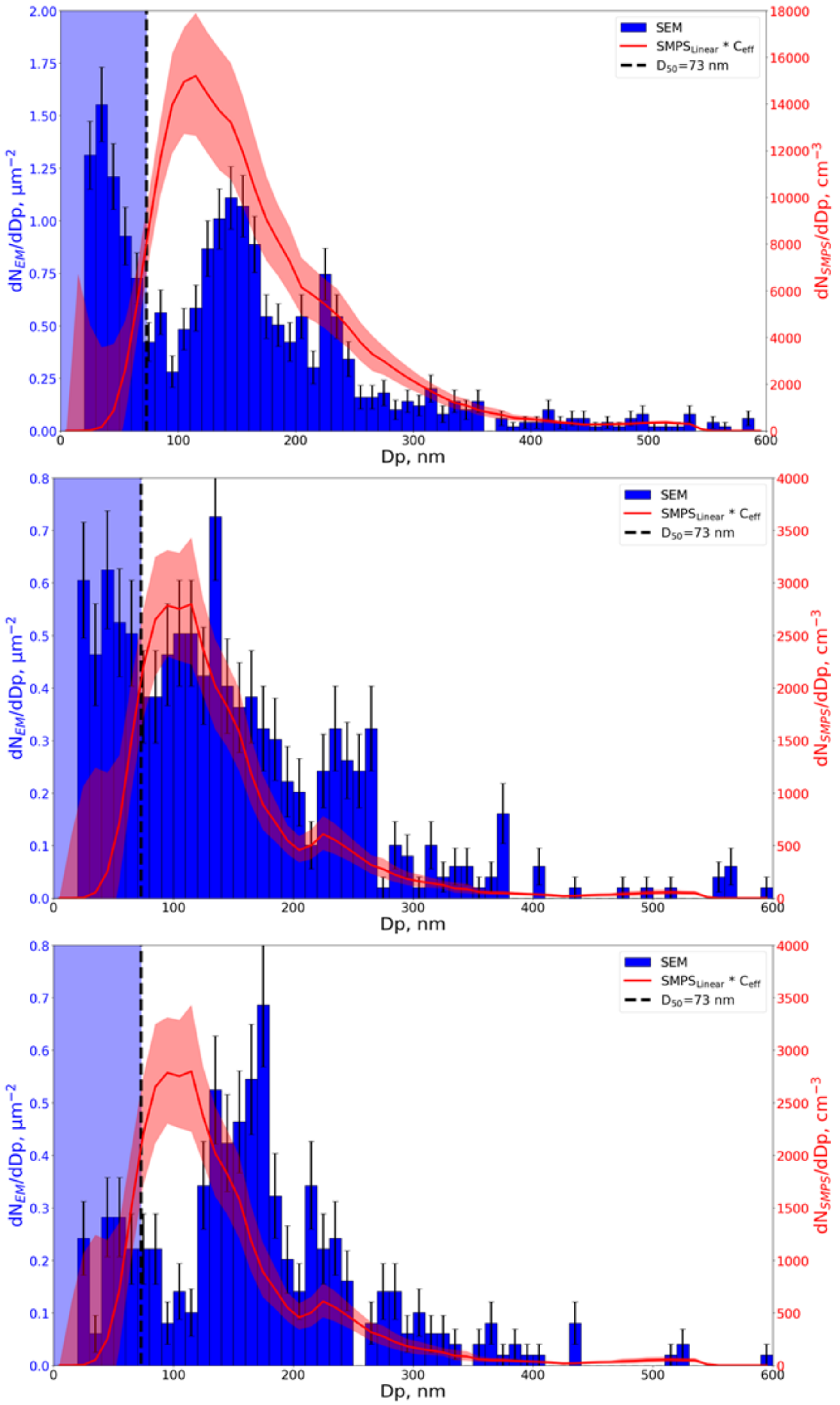

Figure 7. Top: PSD of sample 4 from Table 2. Middle: PSD of sample 4 from Table 2. Bottom:PSD of sample 7 from Table 2

Sampling Time

Measured particle number concentrations, sampling times, number of particles entering the impactor, and sample coverages for the 10 collected impacted aerosols used to determine the optimal collection time are presented in Table 2.

Table 2. Measured particle number concentrations, sampling times, number of particles entering the impactor, and sample coverages for the 10 collected impacted aerosols used to determine the optimal collection time. Abbreviations include the total particle number concentration measured via SMPS C, sampling time with the impactor t, and number of sampled particles Np determined from the Equation found in the main text. Coverage refers to the percentage of the imaged areas recognized as particles.

Sample Number, #	C, cm^{-3}	t, s	Np, #	Coverage, %
1	4.80e5	60	3.6e8	35.9
2	5.10e5	30	1.9e8	40.7
3	5.21e5	20	1.3e8	27.1
4	5.30e5	10	6.7e7	13.9
5	1.00e5	30	3.8e7	14.1
6	8.50e4	20	2.1e7	4.9
7	9.40e4	10	1.2e7	4.1
8	9.40e4	5	6.0e6	3.6
9	1.00e4	30	3.9e6	4.5
10	1.00e4	20	2.5e6	1.6

When visualizing the grids in the SEM it was easily seen that samples 1-3 were overloaded, with very distinctive and highly populated impaction spots. Here the most populated areas were dominated by closely spaced and often very large agglomerates consisting of hundreds of PSL beads. Outside the most populated areas the samples still contained many smaller agglomerates of 10-15 PSL with the number decreasing with increasing distance to the impact center. Samples number 4 and 5 had much fewer and smaller agglomerates in the densely populated areas, typically consisting of a few to tens of PSL beads. In the less populated areas, the samples were dominated by single beads with few agglomerates of 2-5 beads, which could have been airborne as agglomerates. Samples 6-9 had almost no agglomerates in the less populated areas and displayed only very few in the high populated areas, which consisted of only a couple of PSL beads. Sample 10 had much less particles than the previous samples, and displayed only a weak impaction spot, which was barely visible from overview images. However, the sample still contained some agglomerates, indicating that the sampled aerosol contained airborne agglomerates along with the single PSL beads. In general sample 10 had a very low number density, which could make it challenging to do a statistical analysis.

Segmented images of the highest populated areas of samples 1, 4, 6, and 10, as listed in Table 2 are show in Fig. 8. Recognized particles are marked in red, which were used to determine the coverage.

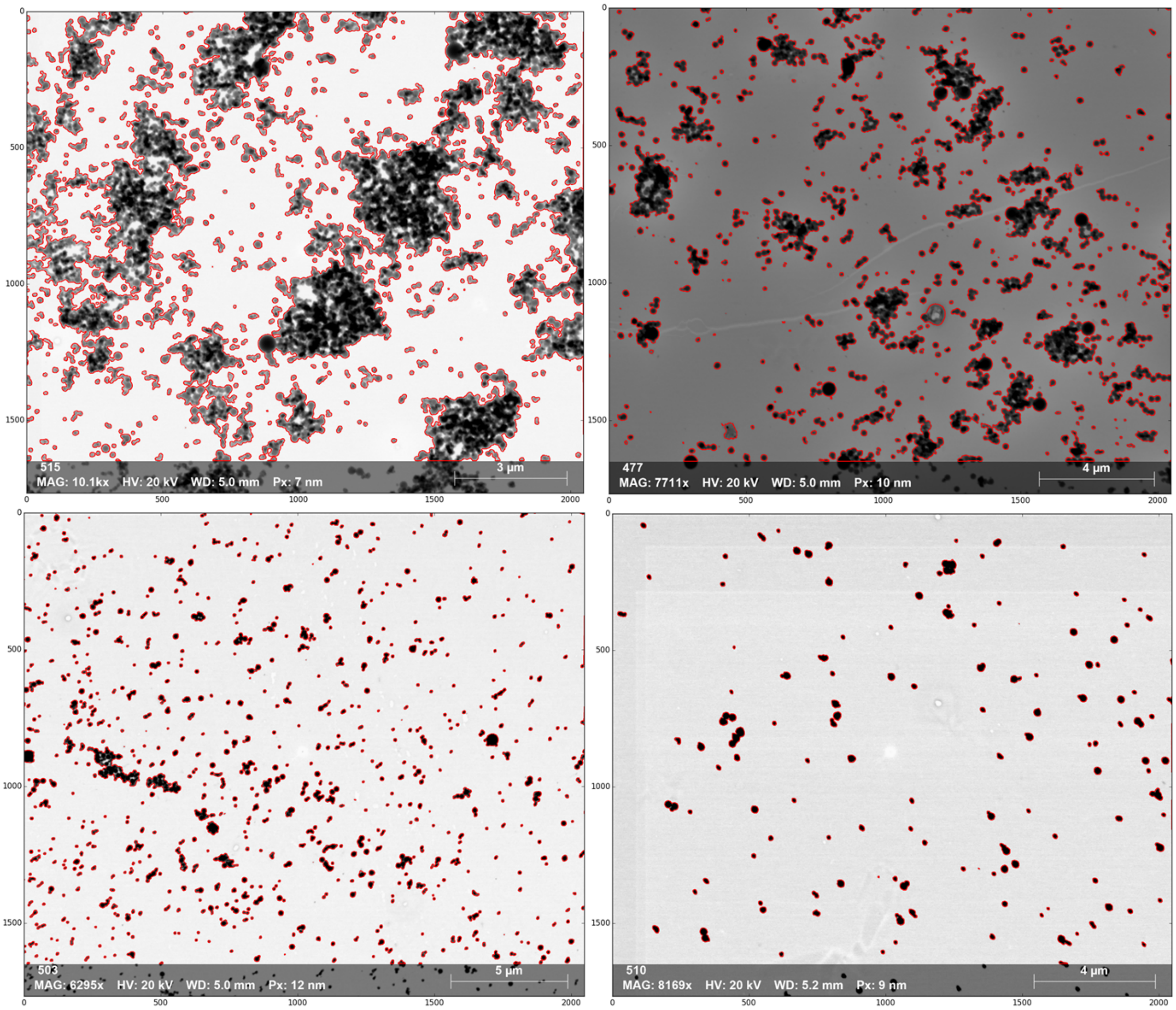

Figure 8. Segmented images of the highest populated areas with recognized particles marked in red, which were used to determine sample coverage. Top Left: Sample 1 from Table 2. Top Right: Sample 4 from Table 2. Bottom Left: Sample 6 from Table 2. Bottom Right: Sample 10 from Table 2.

EDS Examples

Here we present some preliminary automated EDS analysis results from an ongoing study. The sample was collected using the MINI impactor, with the presented results from the third and lowest stage. The sampled aerosol consisted of NaCl particles mixed with Halloysite fibres, which are composed of Si, O, and Al. The NaCl particles were generated with an atomizer, while the Halloysite fibres were aerosolized with a brush generator. The images presented in Fig. 9 include the original secondary electron image (left), the same image with recognized particles classified using a classification scheme, and finally three overlaying EDS maps of Si (cyan), Na (yellow), and Cl (red). The image was segmented using a global threshold. Particles touching the edge of the image frame were discarded as well as particles with an EDS lower than 30 nm. Here the limit of detection could be decreased to 30 nm, since the contrast of the Si and NaCl dominated particles was better than for the PSL, used in the present study. From the images it is seen that automated EDS can be used to successfully distinguish particles of

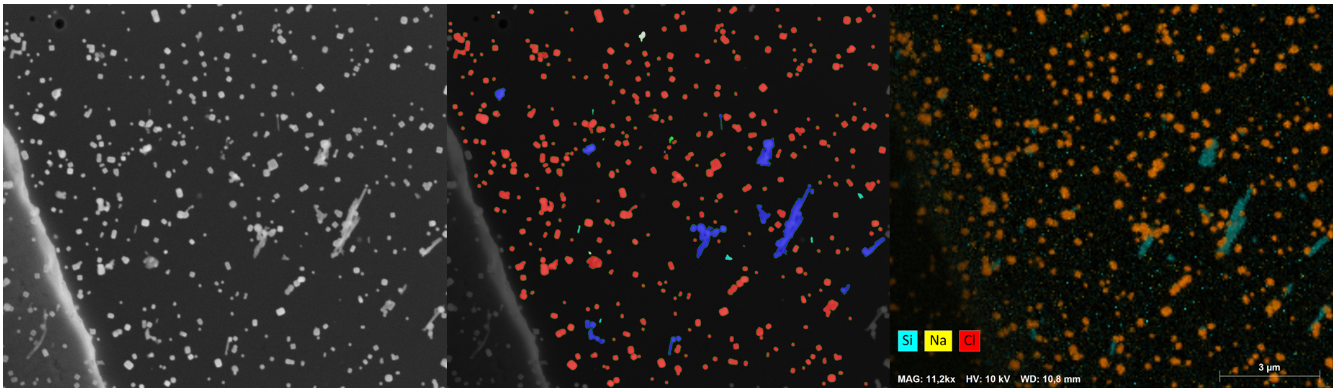

Figure 9. Left: Secondary electron image of the NaCl/Halloysite sample. Middle: Classified image, where pure NaCl particles are marked in red, Halloysite+NaCl particles are marked in blue, pure Halloysite particles are marked in cyan, and white particles are unclassified. Right: Overlaying EDS maps of Si (cyan), Na (yellow), and Cl (red)

different elemental compositions. Furthermore, the use of EDS maps can further distinguish the elemental distribution within a recognized particle, making it possible to determine the mixing state of the particle e.g. homogeneous or phase separated. Combining the EDS results with shape and size data, allows for a thorough and highly detailed characterization of a complex aerosol.

SEM Shape Distributions

Examples of shape distributions are presented as circularity and aspect ratio box plots for the specified size bins. Aspect ratio was determined as the ratio between the longest and shortest dimensions of a rotated bounding box, while circularity was calculated as 4πArea/perimeter^2. It is also possible to plot the particle ECD against the circularity or aspect ratio of the individual particles in a scatter plot. This allows for quick identification of single particles that fulfill the fiber criteria or can be classified as high aspect ratio nanoparticles (HARN). A disadvantage of using the boxplot on the whole particle

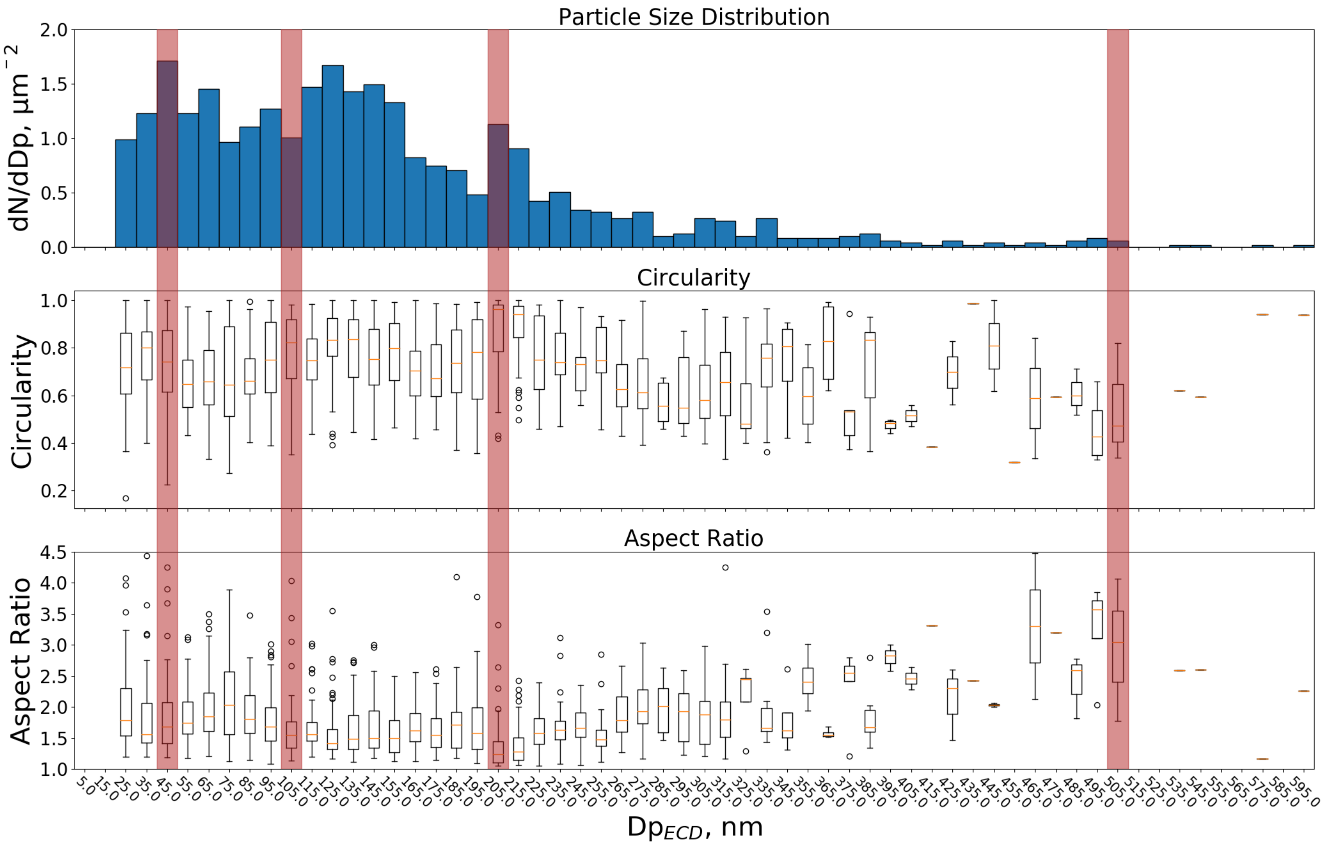

Figure 10. Particle size, circularity, and aspect ratio distribution plots for the dataset from the imaged area marked in red in the Figure found in the main text. The circularity and aspect ratio data are presented as box plots, where the box extends from the lower to upper quartile, with a line at the median. The whiskers extending from the box show the range of the data, while outliers are plotted as individual points. The expected PSL primary sizes of 40, 100, 200, and 500 nm are marked by red shaded areas.

population is that all particles with a given size are pooled together, which can make it difficult to locate high or low aspect ratio particles. An alternative method is to plot a PSD with specifed shape criterias, so that the PSD only includes particles e.g. low circularity. This makes it easier to distinguish primary particle sizes from those dominated by agglomerates. Example of such plots are presented in Fig. 11. Based on the low aspect ratio and high circularity plots, it is evident that the circular shapes and therefore primary particles are dominating for sizes of approximately 40, 100, 140, and 200 nm. These sizes fit well with the expected PSL sizes, which are marked by red shaded areas. The only size not fitting the expected PSL sizes is the 140 nm peak, which may be composed of dense agglomerates, as they can have near circular shapes. For the low circularity and high aspect ratio particles it is seen that the number peaks are located in between the primary PSL sizes, indicating that agglomerates are dominating these sizes. Furthermore it is seen that the number of 100 and 200 nm sizes are much lower than the surrounding, showing that fewer agglomerates were found at the primary PSL sizes.

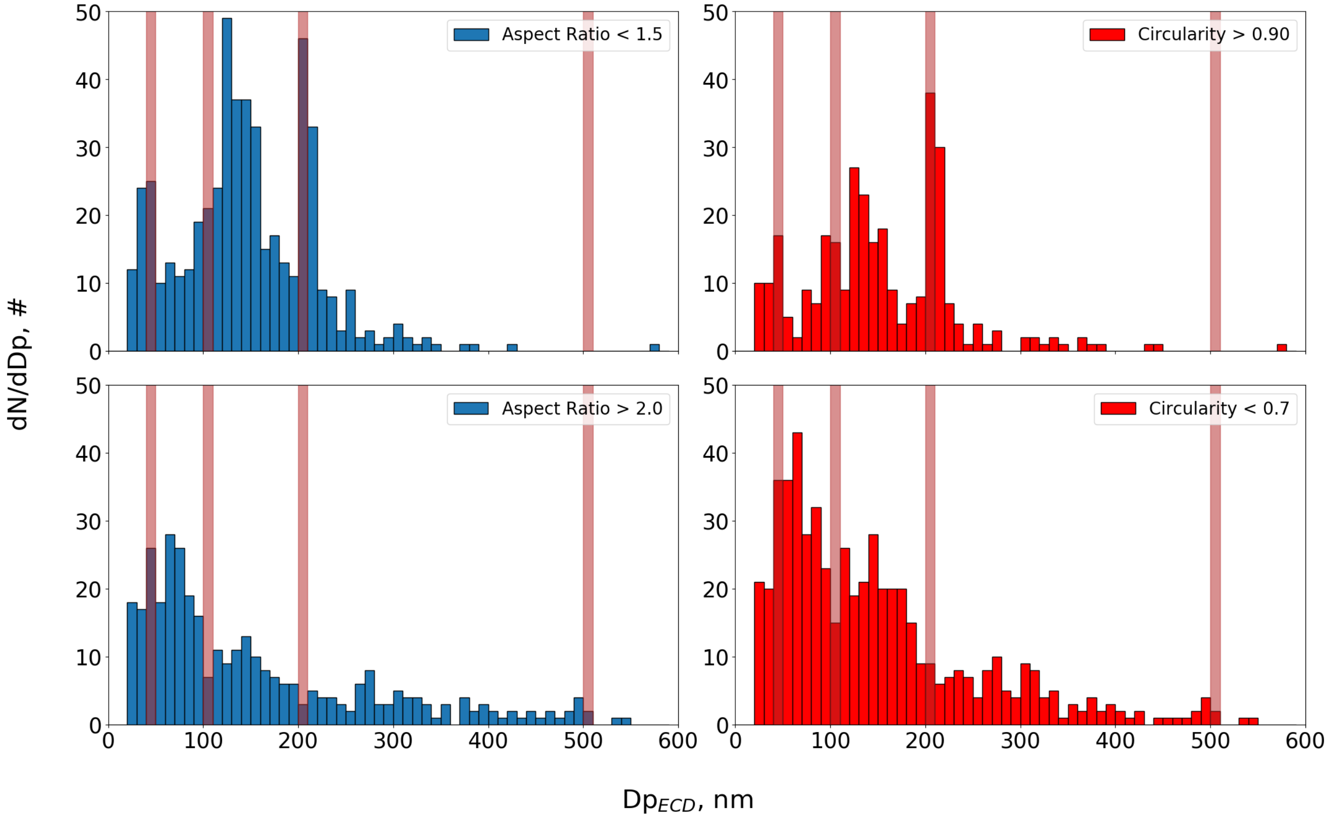

Figure 11. Particle size distribution for particles fulfilling specified shape criteria. Top left: PSD for particles with an aspect ratio lower than 1.5. Top right: PSD for particles with a circularity above 0.9. Bottom right: PSD for particles with an aspect ratio higher than 2.0. Bottom right: PSD for particles with a circularity below 0.7. The expected PSL primary sizes of 40, 100, 200, and 500 nm are marked by red shaded areas.

References

1. Penczek, P. A. Resolution measures in molecular electron microscopy. *Methods Enzymol.* **6879**, DOI: 10.1016/S0076-6879(10)82003-8.Resolution (2011).
2. Hamacher-Barth, E., Jansson, K. & Leck, C. A method for sizing submicrometer particles in air collected on Formvar films and imaged by scanning electron microscope. *Atmospheric Meas. Tech.* **6**, 3459–3475, DOI: 10.5194/amt-6-3459-2013 (2013).
3. Marjamäki, M., Lemmetty, M. & Keskinen, J. ELPI response and data reduction I: Response functions. *Aerosol Sci. Technol.* **39**, 575–582, DOI: 10.1080/027868291009189 (2005).
4. Winklmayr, W., Wang, H. C. & John, W. Adaptation of the twomey algorithm to the inversion of cascade impactor data. *Aerosol Sci. Technol.* **13**, 322–331, DOI: 10.1080/02786829008959448 (1990).
